# Design, optimisation and standardisation of a high‐dimensional spectral flow cytometry workflow assessing T‐cell immunophenotype in patients with melanoma

**DOI:** 10.1002/cti2.1466

**Published:** 2023-09-07

**Authors:** Jack M Edwards, Miles C Andrews, Hayley Burridge, Robin Smith, Carole Owens, Mark Edinger, Katherine Pilkington, Juliette Desfrancois, Mark Shackleton, Sashendra Senthi, Menno C van Zelm

**Affiliations:** ^1^ Alfred Health Radiation Oncology The Alfred Hospital Melbourne VIC Australia; ^2^ Department of Immunology, Central Clinical School Monash University and Alfred Hospital Melbourne VIC Australia; ^3^ Department of Medicine, Central Clinical School Monash University Melbourne VIC Australia; ^4^ Department of Medical Oncology The Alfred Hospital Melbourne VIC Australia; ^5^ Cytek Biosciences Fremont CA USA

**Keywords:** immune checkpoint blockade, immunotherapy, melanoma, spectral flow cytometry, T cells

## Abstract

**Objectives:**

Despite the success of immune checkpoint blockade, most metastatic melanoma patients fail to respond to therapy or experience severe toxicity. Assessment of biomarkers and immunophenotypes before or early into treatment will help to understand favourable responses and improve therapeutic outcomes.

**Methods:**

We present a high‐dimensional approach for blood T‐cell profiling using three multi‐parameter cytometry panels: (1) a TruCount panel for absolute cell counts, (2) a 27‐colour spectral panel assessing T‐cell markers and (3) a 20‐colour spectral panel evaluating intracellular cytokine expression. Pre‐treatment blood mononuclear cells from patients and healthy controls were cryopreserved before staining across 11 batches. Batch effects were tracked using a single‐donor control and the suitability of normalisation was assessed. The data were analysed using manual gating and high‐dimensional strategies.

**Results:**

Batch‐to‐batch variation was minimal, as demonstrated by the dimensionality reduction of batch‐control samples, and normalisation did not improve manual or high‐dimensional analysis. Application of the workflow demonstrated the capacity of the panels and showed that patients had fewer lymphocytes than controls (*P* = 0.0027), due to lower naive CD4^+^ (*P* = 0.015) and CD8^+^ (*P* = 0.011) T cells and follicular helper T cells (*P* = 0.00076). Patients showed trends for higher proportions of Ki67 and IL‐2‐expressing cells within CD4^+^ and CD8^+^ memory subsets, and increased CD57 and EOMES expression within TCRγδ^+^ T cells.

**Conclusion:**

Our optimised high‐parameter spectral cytometry approach provided in‐depth profiling of blood T cells and found differences in patient immunophenotype at baseline. The robustness of our workflow, as demonstrated by minimal batch effects, makes this approach highly suitable for the longitudinal evaluation of immunotherapy effects.

## Graphical abstract

We present an optimised spectral flow cytometry approach for high‐dimensional assessment of T‐cell phenotype in melanoma patients receiving immunotherapy. We showed that this protocol was stable across batches and was capable of detecting an altered systemic immune phenotype in patients. Patients had fewer absolute T cells than controls, mainly due to reduced naive CD4 and CD8 T‐cell numbers and increased proportional expression of Ki67 and IL‐2 across multiple T‐cell subsets.

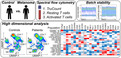

## Introduction

Immune checkpoint blockade (ICB) targeting cytotoxic T‐lymphocyte‐associated protein 4 (CTLA‐4) and/or programmed cell death receptor‐1 (PD‐1) has dramatically improved clinical outcomes for advanced melanoma.^1^, ^2^, ^3^ More recently, lymphocyte activation gene 3 (LAG‐3) inhibitors have entered clinical practice,^4^ with agents targeting additional immune checkpoints in active development. Despite these, most patients still succumb to their disease or experience treatment‐limiting toxicity. To date, no biomarkers have reliably predicted these outcomes in all patients,^5^ including expression of programmed cell death ligand‐1 (PD‐L1) within the tumour microenvironment.^6^

Immune checkpoint blockade modulates the immune response systemically. As peripheral blood is readily and repeatedly accessible during treatment and can reflect intratumoural response,^7^, ^8^, ^9^, ^10^ it likely represents a better opportunity for the discovery of translatable biomarkers than tumour‐based parameters.^11^ Previous studies have suggested a range of immune subsets as putative biomarkers of response or survival during ICB, including the ratio of T‐cell reinvigoration to tumour burden,^7^, ^12^ CD8^+^ T‐cell memory phenotype,^13^, ^14^ natural killer (NK) cell frequencies,^15^, ^16^, ^17^ and the frequency of CD14^+^CD16^−^HLA‐DR^hi^ monocytes.^14^ Broader measures include absolute lymphocyte and eosinophil counts,^18^ as well as the ratio of lymphocytes and monocytes (LMR) or neutrophils and lymphocytes (NLR).^19^, ^20^, ^21^, ^22^ However, in a systematic review of proposed biomarkers, many conflicting results were identified, highlighting the need for further validation studies.^5^ Furthermore, a range of immune parameters have been shown to be important in response to melanoma, including regulatory T cells (Treg), which dampen the immune response and are highly enriched in the blood of melanoma patients,^23^, ^24^, ^25^ while other subsets, such as TCRγδ^+^ (γδT) T cells, can exert both pro‐ and anti‐tumour activity.^26^, ^27^ Thus far, immune cell evaluations have been limited by conventional cytometry and relied on multiple panels to gain phenotyping depth,^13^ only assessed cell subset proportions rather than absolute immune cell counts,^7^, ^28^, ^29^ or suffered from batch effects.^14^ Hence, capturing the complexity of the immune response for biomarker screening and validation of previous findings requires robust and sensitive tools capable of simultaneously measuring a high number of parameters.^30^ Spectral flow cytometry may address these issues with distinct advantages over both conventional flow cytometry and mass cytometry (CyTOF).^31^, ^32^, ^33^, ^34^, ^35^

We here describe our approach to the design, optimisation and analysis of a spectral flow cytometry‐based protocol aimed at discovering markers of response and toxicity to ICB treatment. Our protocol provides absolute counts of innate immune subsets coupled with detailed immunophenotyping of the T‐cell compartment, with the capacity for additional markers to be added as required. Using an exploratory cohort of 15 melanoma patients and 16 age‐matched healthy controls, we demonstrate the capacity of this approach to identify abnormalities in relevant immune cell subsets in melanoma patients.

## Results

### Study subject characteristics

Fifteen patients were included, with a median age of 67 years (range, 56–73), of whom two (13.3%) were female. Blood samples were obtained prior to the start of ICB therapy, which was provided as adjuvant treatment for six patients (40%) and palliative treatment for 9 patients (60%). Sixteen healthy controls were included, with a median age of 63 years (range, 59–72), of whom nine (56%) were female.

### Optimisation of the high‐dimensional spectral flow cytometry panels

**Figure 1:**
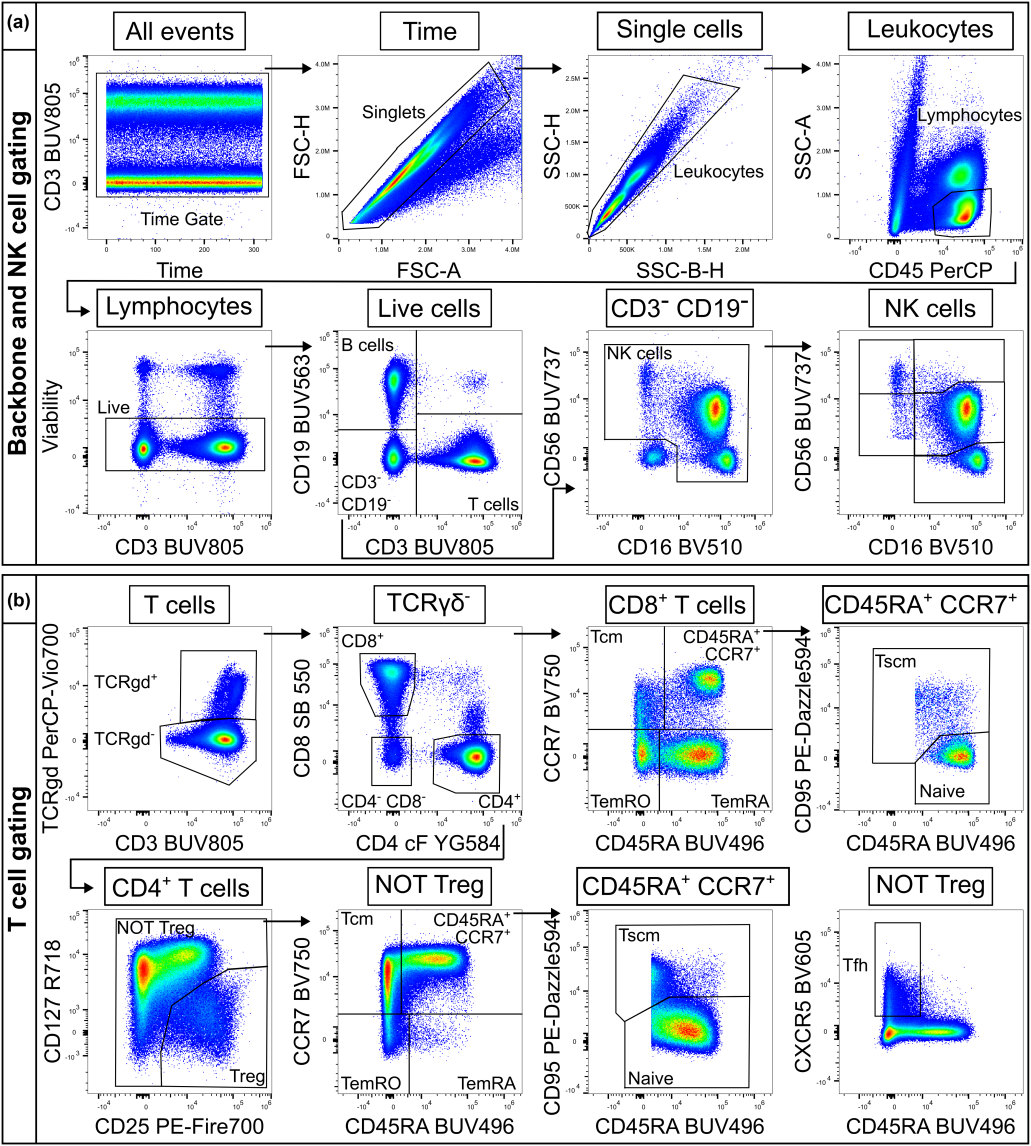
Gating scheme for the 27‐colour resting panel to interrogate T and NK cells for surface and intracellular markers. **(a)** Single, live lymphocytes were gated sequentially before division into B cells (CD19^+^), T cells (CD3^+^) and NK cells (CD19^−^ CD3^−^ CD16/56^+^). NK‐cell subsets were further separated based on differential expression of CD16 and CD56. **(b)** TCRγδ^+^ T cells were defined before CD4 and CD8 lineage gating on the TCRγδ^−^ subset. CD4^+^ Treg cells (CD25^+^ CD127^−/lo^) were excluded before gating memory populations from the NOT Treg gate. Memory populations for both CD4^+^ and CD8^+^ T cells were defined as naive (CD45RA^+^ CCR7^+^ CD95^lo^), stem cell‐like memory (Tscm; CD45RA^+^ CCR7^+^ CD95^hi^), central memory (Tcm; CD45RA^−^ CCR7^+^), effector memory CD45RO (TemRO; CD45RA^−^ CCR7^−^) and effector memory CD45RA (TemRA; CD45RA^+^ CCR7^−^). Circulating CD4^+^ Tfh cells were defined as CD45RA^−^ CXCR5^+^.

**Figure 2:**
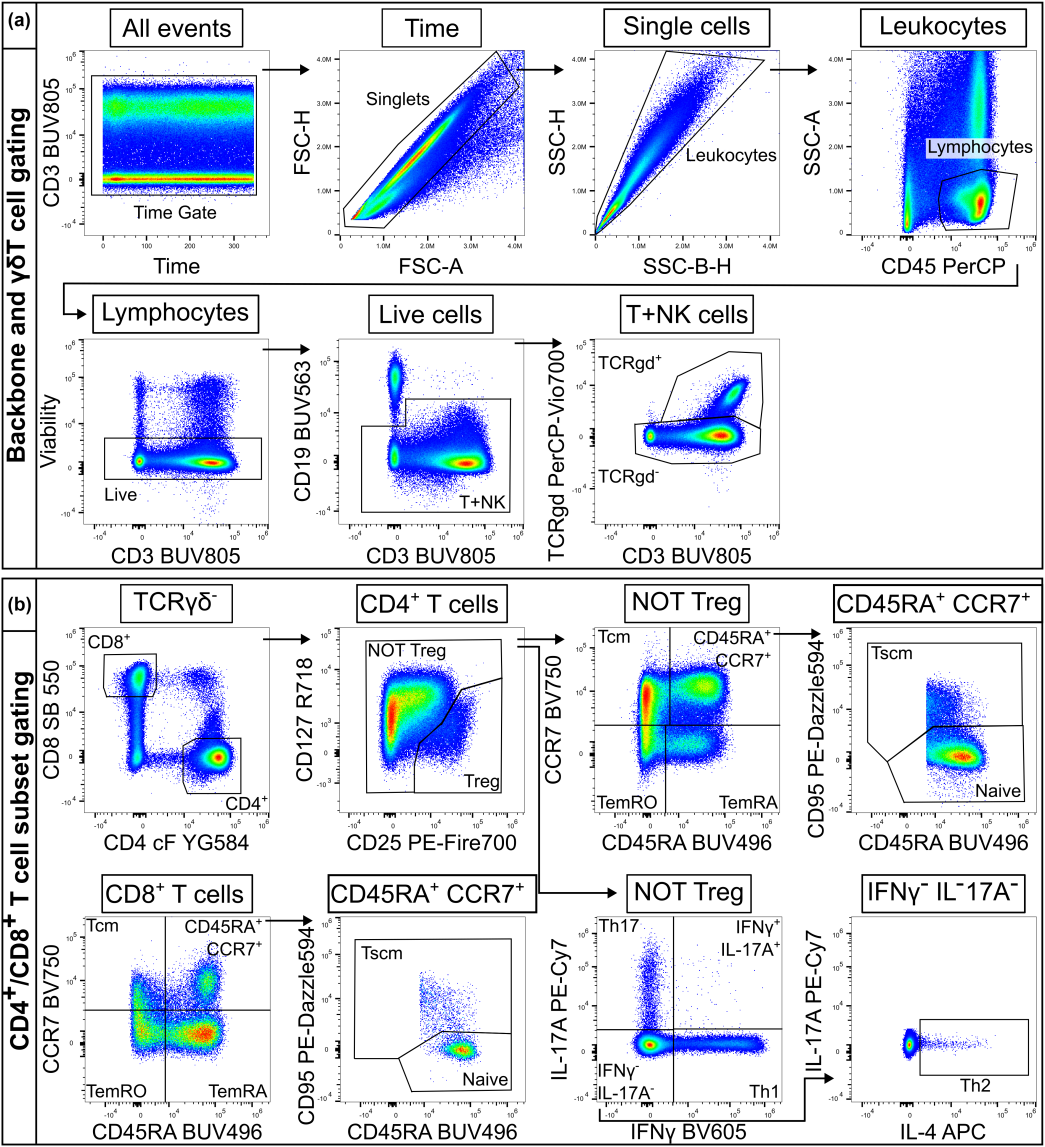
Gating scheme for the 20‐colour activated panel designed to interrogate cytokine production from CD3/CD28‐stimulated T cells. **(a)** Events were gated sequentially to select single, live lymphocytes. Due to CD3 down‐regulation in some samples, T cells were gated from a combined T and NK cell gate (CD19^−^ CD3^+/−^) before gating of TCRγδ^+^ cells. **(b)** CD4^+^ and CD8^+^ T cell subsets were gated as CD19^−^ TCRγδ^−^ CD4^+^ or CD19^−^ TCRγδ^−^ CD8^hi^, respectively (NK cells are TCRγδ^−^ CD8^−/lo^ CD4^−^). Treg were gated as CD25^+^ CD127^−/lo^, with subsequent CD4^+^ T memory and Th‐cell gating from the ‘NOT Treg’ gate. Th cell subsets were defined by canonical cytokine expression (Th1, IFNγ^+^; Th17, IL‐17A^+^; and Th2, IFNγ^−^ IL‐17A^−^ IL‐4^+^). CD4^+^ and CD8^+^ T‐cell memory subsets were defined by differential expression of CD45RA, CCR7 and CD95 as in the resting panel (Figure 1).

Assessment and optimisation of spectral flow cytometry panels for T‐cell immunophenotyping were performed in three consecutive stages: (1) theoretical assessment, (2) antibody titration and (3) experimental assessment of staining, before confirmation of the final panel and staining protocol. Theoretical assessment of marker‐fluorochrome combinations performed using Cytek's similarity and complexity indices^36^, ^37^, ^38^ showed that the 27‐colour resting panel had a complexity index of 10.72 and the 20‐colour activated panel had a complexity index of 7.4 (Table 1, Supplementary figure 1), which compared favourably with similarly large panels.^39^, ^40^ Next, all antibodies were titrated to ensure optimal resolution of markers and the removal of staining artefacts, such as those observed for high concentrations of IL‐17A PE‐Cy7 (Supplementary figure 2). Finally, marker performance in full panel staining was assessed. Comparison of markers to their respective single‐stain reference controls showed minimal loss of resolution in multi‐parameter stains compared to single‐stain controls (data not shown). Assessment of staining patterns demonstrated that the majority of markers performed as expected and reliably identified target lymphocyte populations in both the resting and activated panels, including robust Treg gating using CD25 and CD127 expression (Figures 1 and 2). Two markers required further optimisation. IL‐2 staining was improved after switching from a FITC to an Alexa Fluor 488 conjugate (Supplementary figure 2e). CXCR5 BV605 staining was lost in full panel stains, with potential autofluorescence extraction issues also evident (Supplementary figure 2d). Reagent and protocol alterations were tested, including shifting to a previously validated clone (RF8B2), varying staining temperature (room temperature or 37°C) and intracellular staining. It was determined that a post‐thaw PBMC resting step included in the original protocol (37°C for 2 h before staining) was negatively impacting CXCR5 staining, and removal of this step restored staining to expected levels without affecting any of the other markers (Supplementary figure 2d). Final reagent selections and titres (Supplementary table 1) facilitated reliable identification of target lymphocyte populations in both resting (Figure 1, Supplementary table 2) and activated panels (Figure 2, Supplementary table 3), and the expression of additional classifying markers therein (Supplementary figures 3 and 4).

**Table 1:** TruCount, resting and activated panel markers

Laser	Immunophenotyping panels		
	TruCount (9 parameters)	Resting (27 parameters)	Activated (20 parameters)
UV (355 nm)	–	**Ki67** BUV395	**TNFα** BUV395
	–	**Viability** Live/Dead blue	**Viability** Live/Dead blue
	–	**CD45RA** BUV496	**CD45RA** BUV496
	–	**CD19** BUV563	**CD19** BUV563
	–	**ICOS** BUV661	–
	–	**CD56** BUV737	–
	–	**CD3** BUV805	**CD3** BUV805
Violet (405 nm)	–	**TIGIT** BV421	**IL‐10** BV421
	–	**CD57** Pacific Blue	–
	**CD8** BV510	**CD16** BV510	**Granzyme B** BV510
	**CD4** BV605	**CXCR5** BV605	**IFNγ** BV605
	–	**CCR7** BV750	**CCR7** BV750
	–	**PD‐1** BV786	**CTLA‐4** BV786
Blue (488 nm)	**CD3** FITC	**TIM‐3** BB515	**IL‐2** AF488
	–	**CD8** Spark Blue 550	**CD8** Spark Blue 550
	**CD45** PerCP‐Cy5.5	**CD45** PerCP	**CD45** PerCP
	–	**TCRγδ** PerCP‐Vio700	**TCRγδ** PerCP‐Vio700
Yellow/Green (561 nm)	**CD16 + 56** PE	**Tox** PE	–
	–	**CD4** cFluorYG584	**CD4** cFluorYG584
	–	**CD95** PE‐Dazzle594	**CD95** PE‐Dazzle594
	–	**Tbet** PE‐Cy5	–
	–	**CD25** PE‐Fire 700	**CD25** PE‐Fire 700
	**CD19** PE‐Cy7	**EOMES** PE‐Cy7	**IL‐17A** PE‐Cy7
Red (640 nm)	**HLA‐DR** APC	**KLRG1** APC	**IL‐4** APC
	–	**IRF4** AF647	–
	–	**CD127** R718	**CD127** R718
	**CD14** APC‐H7	**CD39** APC‐Fire750	–

Bold letters denote the target marker, while normal characters denote the fluorochrome.

### Minimal variation between sample acquisition batches

**Figure 3:**
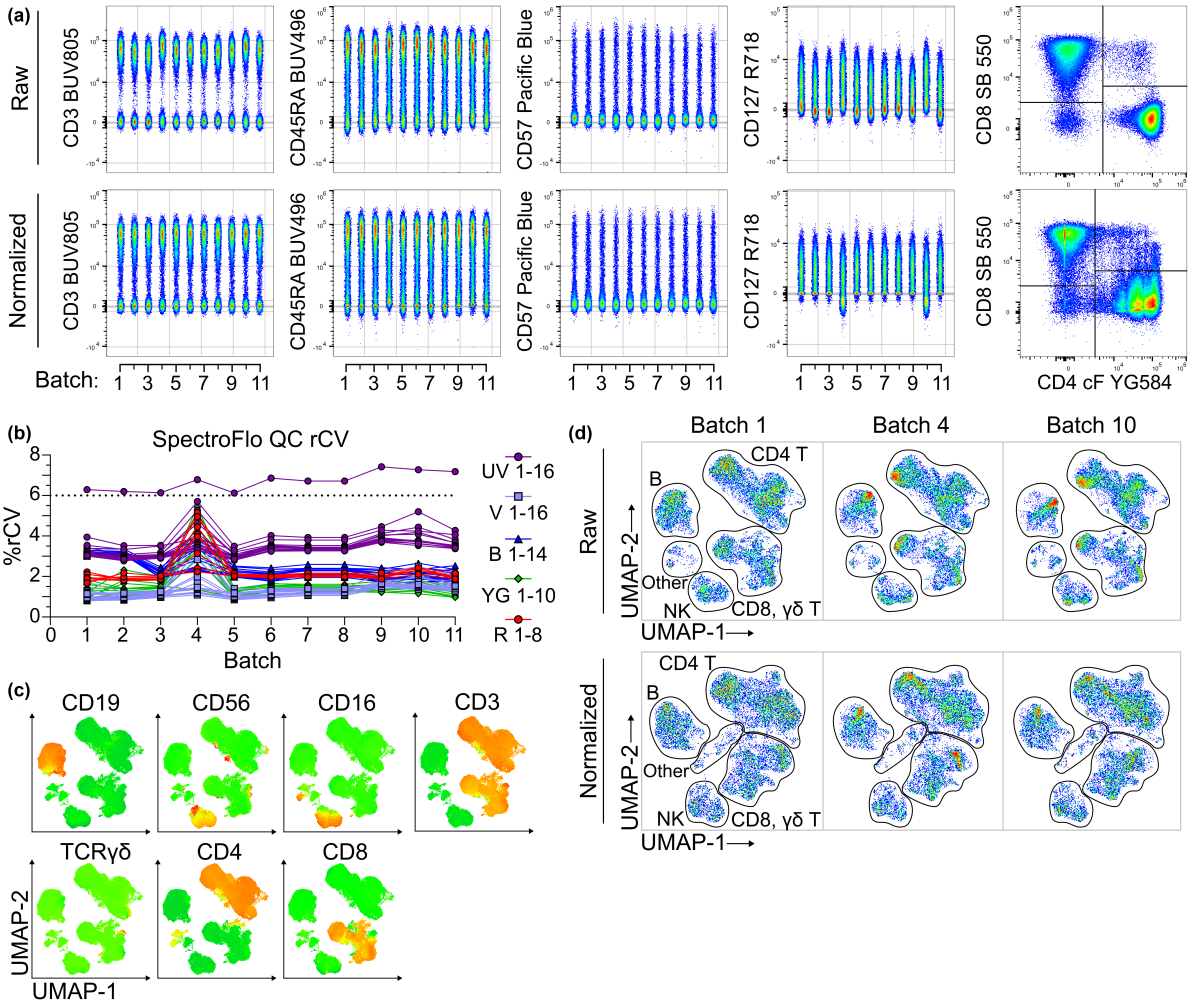
Batch‐to‐batch variation in control data before and after normalisation for batch correction. Batch control data from 11 batches was concatenated and assessed for variation between batches before (‘raw’) and after normalisation (‘normalised’). **(a)** Example of primary (CD3 BUV805), secondary (CD45RA BUV496) and tertiary (CD57 Pacific Blue) marker variation between batches; a marker with more noticeable batch variation (CD127 R718) and manual gating of CD4 cFluor‐YG584 *vs*. CD8 SB 550. **(b)** SpectroFlo daily quality control rCV values indicate underlying cytometer issues for batches 4 and 10. **(c)** Heatmap marker expression overlay for identification of UMAP groups. **(d)** UMAP dimensionality reduction of raw and normalised batch control data for a representative normal plot (batch 1), and batches 4 and 10. CD4^+^ T, CD8^+^ T, NK, B and other populations were assigned manually.

Blood samples in this study were cryopreserved and later thawed and run in 11 separate batches over the course of 3 months, with all instrument reference controls updated after batch 8. To assess the impact of batch variance, aliquots of PBMC from a single buffy coat of the same donor were thawed and stained exactly as for the experimental samples in each batch. Staining remained consistent with only minor variation for markers across batches (example variation shown in Figure 3a; full staining in Supplementary figures 5 and 6). Notably, batches 4 and 10 differed slightly from the other runs (Figure 3a). Daily SpectroFlo QC reports showed that, while not failing the SpectroFlo criteria, %rCV values on the days of acquisition of batches 4 and 10 approached and exceeded the recommended limits for some detectors (Figure 3b). To assess whether high‐dimensional analysis was affected by variation, UMAP dimensionality reduction was performed. UMAP groups were identified and annotated manually and demonstrated reliable grouping of the five major lymphocyte populations: B, NK, CD4^+^ T and CD8^+^ T cells (Figure 3c and d). Comparison of a representative normal batch (batch 1) and batches 4 (black) and 10 (red) showed that these batches remained associated with the correct population groups, indicating that batch effects did not affect global structure (Figure 3d).

However, minor changes in local structure were noted, and to reduce this variation, we applied CytoNorm,^41^ a post‐acquisition data normalisation algorithm. Normalisation of both spectral panels improved the alignment of marker expression across the majority of batches (example plots, Figure 3a; all markers, Supplementary figures 5 and 6). However, CytoNorm introduced artefacts, including compaction of data around 0 and multiple lobes within positive populations, which reduced marker resolution (Figure 3a). Notably, both CD4 and CD8 expression profiles were altered, complicating manual analysis and affecting the global UMAP structure more than any underlying batch‐to‐batch variation, with a loss of distinct separation between CD4^+^ T and CD8^+^ T‐cell populations (Figure 3a and d). Aberrant expression profiles were also noted after normalisation for several additional markers, including CD25 and autofluorescence (Supplementary figure 5b), particularly for the previously mentioned batches 4 and 10. Increasing or decreasing the number of quantiles (default *n* = 101) and/or FlowSOM clusters (default *n* = 10) used by CytoNorm did not correct this issue (Supplementary figure 7). Overall, the results demonstrated that while CytoNorm normalisation was not suitable for this dataset, batch effects were sufficiently negligible that raw data remained suitable for both manual and high‐dimensional analyses.

### Melanoma patients have lower numbers of circulating T cells than controls

**Figure 4:**
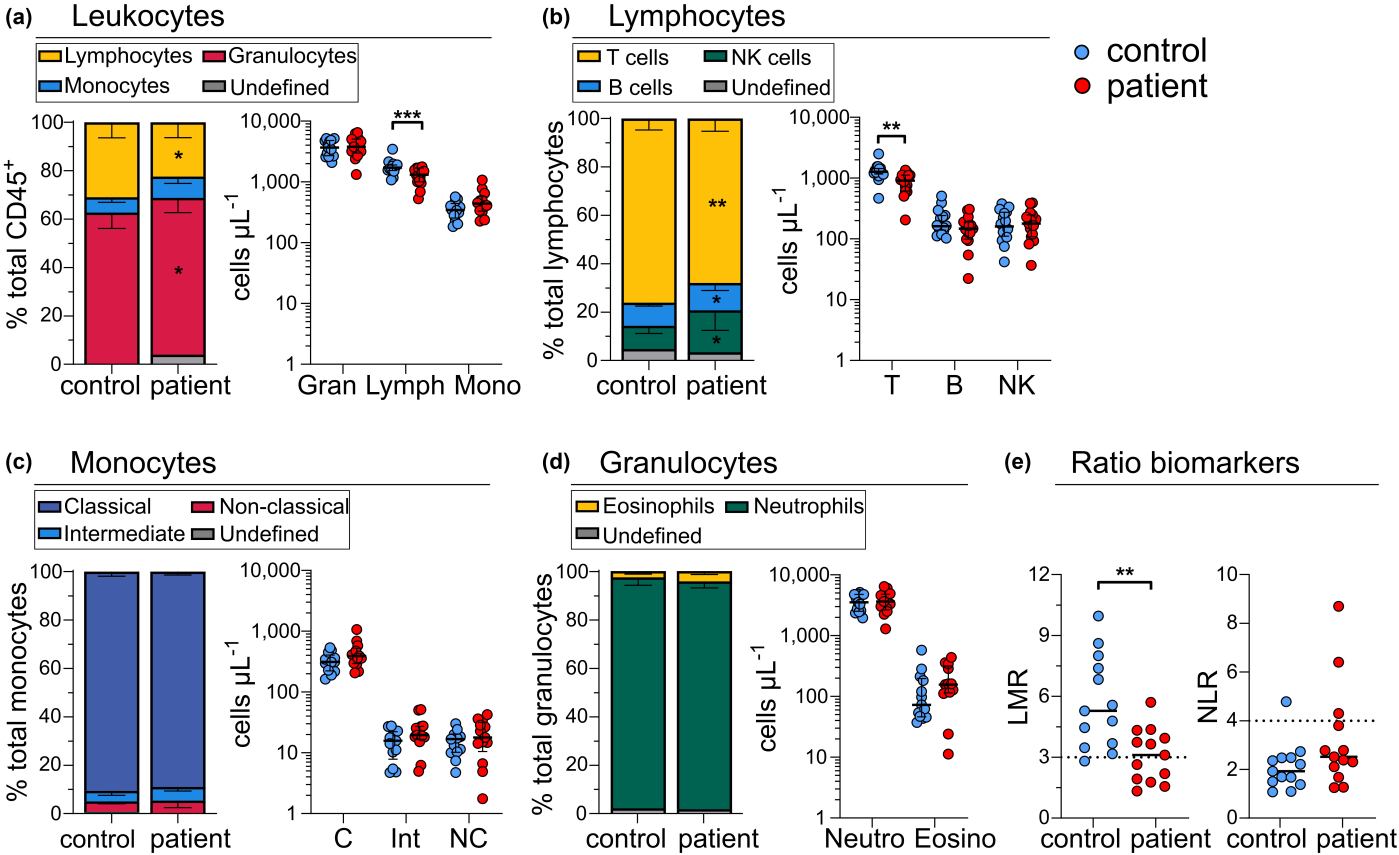
Leukocyte and lymphocyte subset counts in controls and patients with melanoma. Relative (%parent population) and absolute (cells μL^−1^) counts of **(a)** leukocyte (gran: granulocyte, lymph: lymphocyte, mono: monocyte), **(b)** lymphocyte, **(c)** monocyte (C, classical; Int, intermediate; NC, non‐classical) and **(d)** granulocyte (neutro: neutrophil, eosino: eosinophil) subsets. **(e)** Lymphocyte‐to‐monocyte ratio (LMR) and neutrophil‐to‐lymphocyte ratio (NLR). Statistical comparisons between controls (*n* = 15) and patients (*n* = 16) used the unpaired non‐parametric Mann–Whitney test. *P < 0.05, **P < 0.01, ***P < 0.001. Results show median values ± interquartile range. Stacked relative frequency bar plots were adjusted to sum to 100% with the addition of the ‘undefined’ population.

Absolute immune cell counts from TruCount data showed that patients had significantly fewer lymphocytes at baseline than healthy controls (median, 1313 cells μL^−1^ *vs*. 1715 cells μL^−1^; *P* = 0.0027) (Figure 4a), which was predominantly due to fewer T cells (median, 907 cells μL^−1^ *vs*. 1271 cells μL^−1^; *P* = 0.0015) (Figure 4b). No significant differences were observed for total leukocyte counts between patients and controls (median, 5953 cells μL^−1^ and 5835 cells μL^−1^, respectively). Similarly, total granulocyte and monocyte counts (Figure 4a), as well as derivative subsets, i.e. classical, intermediate and non‐classical monocytes (Figure 4c) and neutrophils and eosinophils (Figure 4d **)**, were equivalent between patients and controls. Total B cell and NK cell counts were equivalent between the groups, indicating that the significant increases in B and NK cell frequencies were due only to a decrease in T cells (Figure 4b). Lower patient lymphocyte numbers also contributed to a decreased LMR ratio (Figure 4e).

### Differences in patient and control T‐cell populations are visualised by semi‐unsupervised high‐dimensional analysis

**Figure 5:**
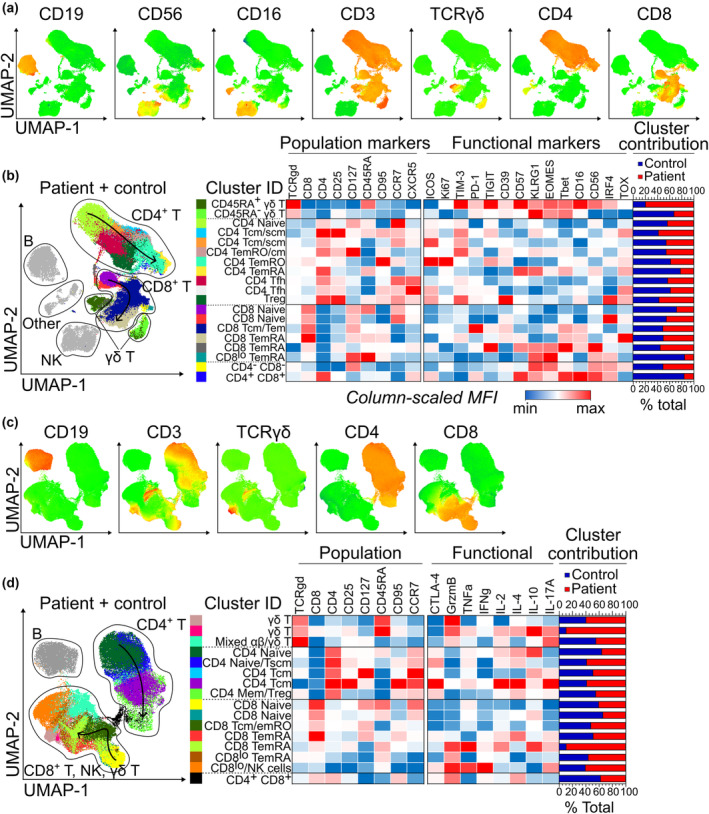
High‐dimensional analysis shows reduced naive T‐cell subsets and altered γδT‐cell phenotype in melanoma patients. **(a)** Resting panel UMAP plots with heatmap overlay of population marker expression. **(b)** X‐shift clustering overlay of combined control and patient resting panel data with column‐scaled median fluorescence intensity (MFI) of all markers and percent contribution of patients and controls to each cluster. Clustering was performed on T cells using the population markers denoted in the heatmap. **(c)** Activated panel UMAP plots with heatmap overlays of population marker expression. **(d)** X‐Shift clustering overlay and median expression for activated panel clusters. Manually drawn arrows on X‐shift plots represent the progression of CD4 and CD8 T cells from naive to terminally differentiated effector memory.

To assess the suitability of high‐dimensional analysis for initial data exploration and identification of target populations, UMAP dimensionality reduction and X‐Shift clustering were performed. Heatmap overlays of individual marker expression on UMAP plots were used to identify major populations (Figure 5a); for the resting panel data, UMAP successfully separated NK, B, γδT, CD4^+^ T and CD8^+^ T cells (Figure 5a and b). Activated panel data showed similar results except for a combined CD8^+^ T, γδT and NK cell group, highlighting the effect of a lack of distinct NK‐cell markers in this panel and downregulation/masking of CD3 after stimulation (Figure 5c and d).

To interrogate T‐cell phenotype, X‐Shift clustering was performed on manually gated T cells (resting panel) or T and NK cells (activated panel). 19 clusters were identified for the resting panel and 16 clusters were identified for the activated panel; these clusters were overlaid onto UMAP plots and putative cluster identities assigned based on a manual gating strategy (Figure 5b and d). Resting panel clustering identified two distinct γδT‐cell populations (CD45RA^±^), CD4^+^ Tfh and Treg, and CD4^+^ and CD8^+^ naive T‐cell subsets. CD4^+^ and CD8^+^ T‐cell memory clusters did not precisely relate to manually gated populations, likely due to the expression patterns of the relevant markers (CCR7, CD45RA and CD95). However, clustering identified general stages of memory differentiation from naive to terminally differentiated effector memory, and these populations were positioned sequentially on UMAP plots (denoted by manually drawn arrows; Figure 5b and d). Activated panel clustering was similar, except for an additional mixed αβ/γδT‐cell population (defined by manual gating), a mixed Treg/CD4 memory population and a CD8^lo^/NK cell cluster.

Heatmaps showing per‐marker column‐scaled median fluorescence intensity and the relative contribution of patient and control groups to each cluster were used for further phenotypic exploration of clusters (Figure 5b and d). Controls accounted for a greater proportion of the abundant CD4 naive cluster (57%) and joint CD8 naive (62%) clusters (Figure 5b). Additionally, patient γδT cells appeared skewed towards an activated phenotype expressing high levels of inhibitory and differentiation markers, including TIM‐3, TIGIT, CD57, KLRG1, EOMES and CD56, as well as granzyme B; patients accounted for 79% of this CD45RA^+^ γδT cluster (Figure 5b). Both panels generated data suitable for high‐dimensional analysis, which provided a useful qualitative ‘first look’ at the data and highlighted several populations of interest for validation in manual analysis.

### Reduced CD4 and CD8 naive T‐cell numbers in melanoma patients

**Figure 6:**
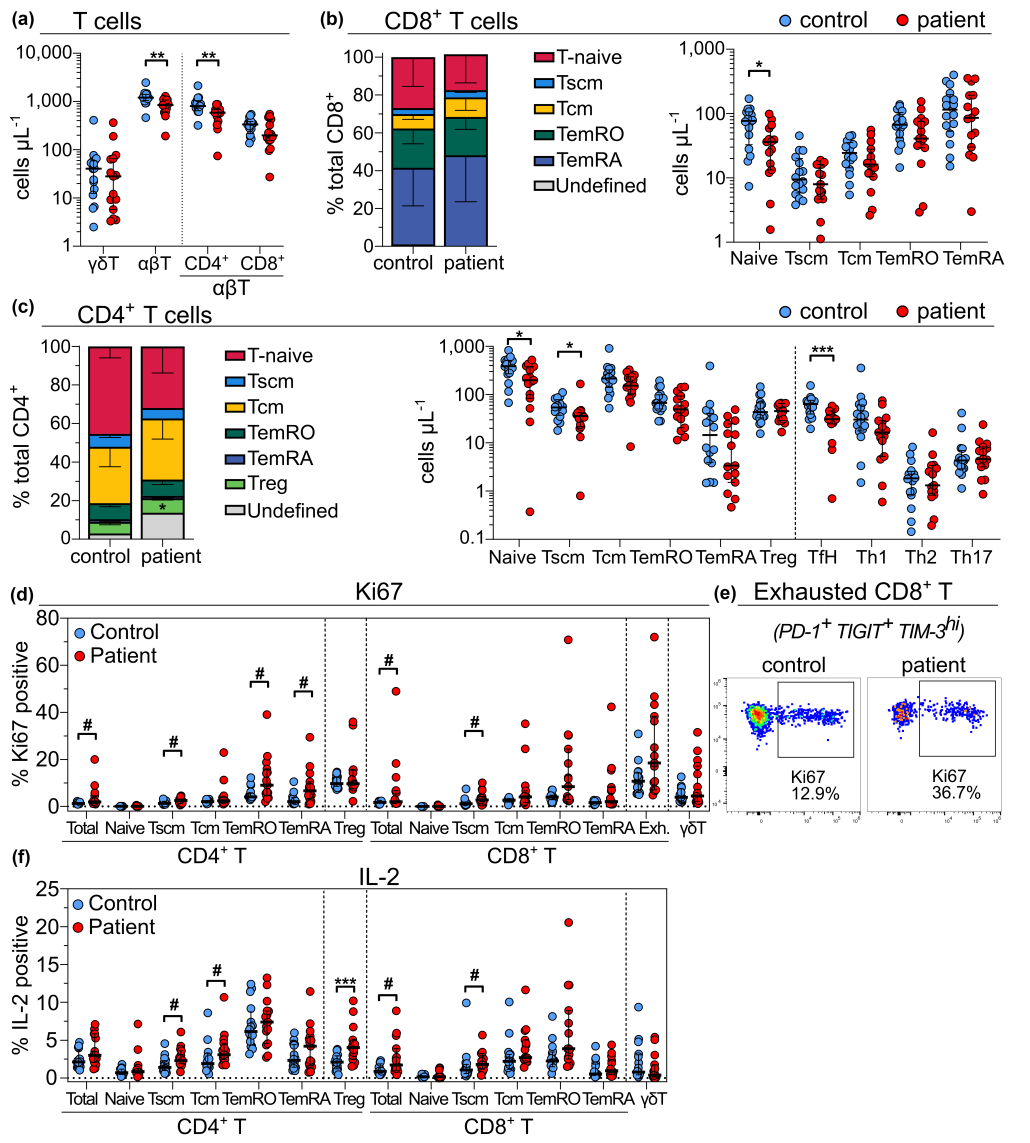
Resting and activated panels facilitate in‐depth immunophenotyping of the T‐cell compartment in melanoma patients. **(a)** Absolute counts of major T cell subsets show reduced abundance of αβT cells in patients, mostly attributed to a decrease in CD4 T cells. Relative and absolute counts of CD8 **(b)** and CD4 **(c)** T cell subsets demonstrate a significant reduction of naive and Tfh cells. **(d)** Differential Ki67 expression in CD4 and CD8 T‐cell subsets, including exhausted (Exh.) PD‐1^+^ TIGIT^+^ TIM‐3^hi^ CD8 T cells. **(e)** Example Ki67 staining in exhausted CD8 T cells **(f)** Differential IL‐2 expression in CD4 and CD8 T cell subsets. All statistical comparisons used the unpaired, non‐parametric Mann–Whitney test. *P < 0.05, **P < 0.01, ***P < 0.001. Hash (#) symbols denote changes that did not reach significance after Bonferroni multiple comparisons correction. Results are shown as median ± interquartile range. Stacked relative frequency bar plots were adjusted to sum to 100% with the addition of the ‘undefined’ population.

Manual analysis of the data showed that the decreased T‐cell numbers observed in patients were principally due to reduced conventional TCRαβ^+^ (αβ)T cells (median, 847 cells/μL in patients *vs*. 1208 cells μL^−1^ in controls; *P* = 0.00044), while γδT cells were not significantly different (Figure 6a). Within the αβT‐cell compartment, patients displayed a decrease in CD4 T cells (*P* = 0.0017), largely due to the naive subset (median, 199 cells μL^−1^ *vs*. 389 cells μL^−1^; *P* = 0.015), as well as CD4 Tscm (*P* = 0.015) and Tfh (*P* = 0.00076) cells. Naive CD8 T‐cell numbers were also lower in patients (median 36 cells μL^−1^ *vs*. 77 cells μL^−1^; *P* = 0.011). No significant differences were observed in the absolute numbers of Treg, T helper (Th) or other memory subsets within the CD4 and CD8 lineages, despite an apparent proportional increase in Treg cells (Figure 6b and c). After correcting for multiple comparisons in major T cell lineages (γδT, CD4 and CD8), the absolute decrease in CD4 T cells remained significant.

Together, the resting and activated panels allowed the expression of 18 markers to be quantified on T cell subsets, highlighting the capacity of the developed panels to assess a range of markers relevant for response to ICB (Figure 6d–f, Supplementary figure 8a–c). *P‐*values were adjusted for multiple comparisons using the Bonferroni method (18 markers; adjusted *P*‐value cut‐off = 0.0028). Comparisons that did not reach significance after adjustment are denoted by a hash (#) symbol within figures (Figure 6). A significantly higher proportion of patient Treg cells expressed IL‐2 (*P* = 0.0012), while there were also trends noted for increased IL‐2 expression in patient CD4 Tscm (*P* = 0.021) and Tcm (*P* = 0.024), and CD8 total (*P* = 0.03) and Tscm (*P* = 0.027) that did not reach significance after multiple comparisons adjustment (Figure 6f). Similarly, a trend towards increased Ki67 expression was noted for more CD4 total (*P* = 0.011), Tscm (*P* = 0.024), TemRO (*P* = 0.027) and TemRA (*P* = 0.0082), and CD8 total (*P* = 0.045) and Tscm (*P* = 0.0055); however, these differences did not reach significance after adjustment (Figure 6d). Patient and control total CD8 and CD4 T cells showed no other significant differences in the expression of the remaining inhibition, differentiation and cytokine markers (Supplementary figure 8a and b).

Co‐expression of multiple inhibitory markers is indicative of immune exhaustion.^42^ Boolean combinatorial gating of the inhibitory markers TIM‐3^hi^, PD‐1 and TIGIT on non‐naive CD8^+^ T cells (‘exhausted’ cells; defined as CD8^+^ CD45RA^+/−^ CD95^+^) showed no significant differences in co‐expression patterns between patient and control groups (Supplementary figure 8d). Furthermore, the co‐expression patterns of multiple cytotoxic markers (IFNγ, Granzyme B, TNFα and IL‐2) by CD8^+^ non‐naive T cells were not significantly different (Supplementary figure 8e). However, patients tended to have a higher proportion of cells positive for all three exhaustion markers that expressed Ki67 (*P* = 0.06) (Figure 6d and e).

Following high‐dimensional analysis (Figure 5), further exploration of γδT cell phenotype demonstrated that patients trended towards increased proportional expression of CD57 (*P* = 0.041) and EOMES (*P* = 0.033). A decreased proportion of patient γδT cells expressed IL‐17A (*P* = 0.027) and TNFα (*P* = 0.047), but differences to other markers indicated by high‐dimensional analysis were not statistically significant in manual analysis (Supplementary figure 8c).

Overall, in comparison to healthy controls, melanoma patients demonstrated reduced numbers of CD4 and CD8 naive T cells coupled with indications of memory T‐cell activation denoted by increased Ki67 and IL‐2 positivity and a skewed γδT‐cell phenotype.

## Discussion

Here, we describe the design, optimisation and initial application of a comprehensive high‐parameter spectral flow cytometry protocol for the evaluation of T‐cell changes in patients with solid malignancies. Our approach reliably identified major immune subsets and provided detailed immunophenotyping of the T‐cell compartment capable of detecting differences between baseline patient samples and age‐matched healthy controls. Notably, this protocol facilitates the concurrent assessment of multiple previously proposed markers of response to ICB and retains the capacity for additional markers to be added as required. Additionally, the inclusion of absolute cell counts performed at the time of sampling removes reliance on relative frequencies, which may otherwise lead to misleading results. Thus, this optimised approach will be suitable for the identification of biomarkers to predict the outcome of ICB before or early after commencement of treatment; and this pilot study will inform future analysis strategies and enable accurate statistical power calculations.

High‐parameter single‐cell evaluations are necessary to capture the complexity of the immune system in the context of a malignancy and after the commencement of ICB treatment. Spectral flow cytometry offers advantages such as an increased number of measurable parameters, improved sensitivity of detection compared to conventional cytometry^32^, ^36^ and higher event rates, sensitivity and accessibility than CyTOF.^33^, ^34^, ^35^ The resting and activated T‐cell panels developed here allowed reliable evaluation of 18 markers across 15 T‐cell subsets, facilitating detailed evaluation of the T‐cell compartment and generating data well suited to high‐dimensional data analysis.

Our evaluation of 15 pre‐treatment patients and 16 age‐matched healthy controls revealed that patients had fewer lymphocytes, predominantly due to lower CD4 and CD8 naive T‐cell numbers and CD4 Tfh cell numbers (Figure 6a–c). Similar findings of proportionally reduced total, CD4 T and CD8 naive T cells have been reported in cohorts of stage IV melanoma,^14^ and reduced absolute counts of total, CD4 T and CD8 T cells in non‐small cell lung cancer (NSCLC).^13^ A higher proportional Treg abundance has previously been reported in melanoma patients^7^; however, our evaluation of absolute cell counts demonstrated that this may have been due to decreases in other CD4 T‐cell subsets (Figure 6c), highlighting the pitfalls of relying on proportional frequencies. Remaining granulocyte, monocyte, B, NK, γδT and regulatory and memory T‐cell subsets were present at similar levels in both groups. However, high‐dimensional evaluation revealed that patient γδT cells were skewed towards a CD57^+^ terminally differentiated effector phenotype (Figure 5b), a finding that was validated by our manual analysis (Supplementary figure 8c) and in line with recent findings.^43^ Manual validation of high‐dimensional analyses remains important, as common data visualisation tools such as heatmap expression plots are affected by the choice of scaling,^44^ and median expression levels may not accurately represent marker expression profiles. Furthermore, these visualisation tools must be interpreted in light of the known biological significance of marker expression level, which may be continuously related to expression for some markers while displaying threshold effects for others.^45^, ^46^

High‐dimensional evaluation of large datasets by flow cytometry has previously been hampered by technical variation between experiments, an issue termed ‘batch effects’.^47^, ^48^ Previous studies assessing batch effects have typically focused on shorter time periods, fewer batches or utilised CyTOF,^41^, ^49^, ^50^, ^51^ making this study notable for its exploration of longer term batch effects as they apply to spectral flow cytometry. The robustness of our protocol for longitudinal immunophenotyping was demonstrated by stable staining performance over the 3‐month time interval during which batches were run (Figure 3). These results suggest that with adequate standardisation of both the experimental protocol and daily cytometer performance, single‐centre studies such as this can reduce significant batch effects and generate data suitable for longitudinal studies. In addition, protocol and instrument standardisation avoids the need to use post‐acquisition normalisation algorithms, which can introduce artefacts that can negatively impact both manual and high‐dimensional analyses (Figure 3). While the minor batch variation observed here may be due to several issues,^47^ we postulate that it predominantly arose from isolated cytometer issues that were not adequately addressed by SpectroFlo QC. Interestingly, we noted that minor post‐acquisition compensation adjustments were able to partially rescue these variant batches.

After full analysis of our cohort, it was evident that several limitations to the panels remained, notably CD3 downregulation or masking after stimulation (Supplementary figure 6a) and TOX‐PE staining, which was included in the resting panel despite suboptimal performance as it did not affect the resolution of other markers. TOX‐PE staining may have been impacted by CD4 cFluor‐YG584, which peaks in the same detector. Intracellular staining of CD3 after stimulation and shifting TOX to a more appropriate fluorochrome will address these issues.

We have here explored the development and initial application of a robust spectral flow‐cytometric approach to immunophenotyping in patients undergoing ICB. Our protocol enabled consistent staining performance over multiple batches and demonstrated notable baseline differences between patients receiving ICB and age‐matched healthy controls. The inclusion of multiple previously suggested markers of response and toxicity to ICB into one protocol, as well as the ability to expand the panel if required, positions this protocol as an excellent approach for the discovery of biomarkers that accurately predict treatment outcome before or early after the commencement of ICB therapy.

## Methods

### Patients and healthy volunteers

Patients commencing ICB‐containing systemic therapy regimens for the treatment of completely resected (adjuvant) or advanced, unresectable melanoma were eligible for inclusion and recruited through The James Foster Foundation Alfred Cancer Biobank under protocols approved by the Alfred Ethics Committee (AEC 535/19). Written informed consent was obtained from all participants. Stored blood samples were retrieved under the registry‐based observational study Translational Research in Immunotherapy Patients (TRIP; AEC 507/20). Age‐matched healthy controls were recruited under an approved protocol from Monash University (26385), and blood samples were collected after obtaining written informed consent. All studies were conducted in accordance with the ethical principles of the Declaration of Helsinki and with adherence to the Good Clinical Practice guidelines as defined by the International Conference on Harmonisation.

### Blood sample processing

Blood samples were collected in Vacutainer K2EDTA blood tubes (BD Biosciences, San Jose, CA). Total leukocyte counts were determined with the Cell Dyn analyser (Abbott Core Laboratory, Abbott Park, IL, USA), and in addition, 50 μL was taken for flow‐cytometric TruCount analysis (see below). Blood samples were centrifuged at 1320 *g* for 10 min, plasma was removed and centrifuged again at 12 000 *g* for 10 min before storage at −80°C. The remaining blood was diluted 1:1 with phosphate‐buffered saline (PBS) and loaded onto Ficoll–Hypaque Plus (Cytiva, Marlborough, MA, USA) for isolation of peripheral blood mononuclear cells (PBMCs) by density centrifugation at 650 *g* for 25 min with no brake. After washing twice with PBS, PBMCs were cryopreserved in liquid nitrogen at a density of 10 million viable cells mL^−1^ in 50% RPMI‐1640 (Sigma‐Aldrich, St. Louis, MO, USA), 40% FCS (Bovogen, Melbourne, VIC, Australia) and 10% DMSO (Sigma‐Aldrich).

Batch and reference control samples: PBMCs from a single buffy coat pack were isolated as described above, except that blood was diluted and washed with heparinised medium (RPMI‐1640 + 0.3 g L^−1^ L‐glutamine, 1% penicillin–streptomycin and 10 IU mL^−1^ heparin) before cryopreservation at either 50 million cells/vial for reference controls or 5 million cells/vial for batch controls.

### TruCount analysis

Absolute numbers of leukocytes were determined using a lyse‐no‐wash T, B and NK TruCount assay, as previously described,^52^ with the inclusion of CD14 and HLA‐DR to facilitate enumeration of granulocyte and monocyte subsets (Supplementary figure 9). Within 6 h of sample collection, 50 μL of whole blood was added to a TruCount tube (BD Biosciences) together with an antibody cocktail of 20 μL containing conjugated primary antibodies against human CD3, CD4, CD8, CD14, CD16, CD19, CD45, CD56 and HLA‐DR (Table 1, Supplementary tables 4 and 5). Following incubation for 15 min at room temperature, samples were lysed with FACS Lysing Solution (BD Biosciences) in a total volume of 500 μL for 15 min at room temperature and stored at 4°C in the dark for up to 1 h before acquisition on an LSRII or FACSLyric flow cytometer (BD Biosciences).

### Spectral flow panel design

Based on previous work^53^ and according to established spectral panel design principles,^36^ two spectral flow panels were designed: (1) a resting panel targeting both surface and intracellular markers expressed on *ex vivo* resting T cells and (2) an activated panel targeting intracellular cytokine production after overnight CD3 and CD28 stimulation (Table 1, Supplementary table 1). Briefly, spectrally distinct fluorochromes were selected based on brightness and spread to minimise panel complexity calculated using the Cytek Similarity and Complexity Index.^38^ Marker‐fluorochrome combinations were selected to pair dimly expressed antigens with bright fluorochromes and *vice‐versa* to ensure adequate resolution while minimising spread. In addition, spectrally distinct fluorochromes were selected for co‐expressed antigens to further limit spread. Where possible, previously validated clones were selected.^53^ In the case of IL‐4, the initially tested clone 8D4‐8 was exchanged for MP4‐25D2, which resulted in improved binding specificity,^54^ and more reliable performance. UltraComp eBeads (Invitrogen, Waltham, MA, USA) were used in reference controls for IL‐2 AF488, IL‐4 APC, IL‐10 BV421 and IL‐17A PE‐Cy7. All remaining reference controls used single‐stained cells from a single donor, whereas unstained controls were generated for each donor and staining condition. Reference controls were updated after approximately 3 months; when new antibody lot numbers were used, these were first tested on PBMCs from the same donor sample as the original.

### Antibody titration for spectral flow panels

Antibody titrations were performed on thawed PBMCs under staining conditions matching those used in full panel stains. Antibodies targeting primary antigens, defined as those with clear positive and negative expression patterns (typically major lineage markers such as CD3, CD4, CD8, CD16, CD19 and CD56), were titrated alone, and emission data in the relevant peak channel were used for analysis. Antibodies targeting secondary and tertiary antigens, displaying continuous or low expression levels, respectively, were titrated in the presence of lineage markers to enable evaluation within the populations of interest. Titrations were started at twice the concentration recommended by the manufacturer and continued with 2‐fold dilutions over a 6‐point series. Optimal titres were defined by the maximum separation of positive and negative populations using a separation index calculated as

$$\frac{{\text{median}}_{\mathrm{pos}}-{\text{median}}_{\mathrm{neg}}}{\left(\frac{84^{\mathrm{th}}\;\text{percentile negative}-{\text{median}}_{\mathrm{neg}}}{0.995}\right)},$$

where median_pos_ and median_neg_ represent the median fluorescence intensity of manually gating positive and negative marker populations, respectively (Supplementary figure 2a–c).^55^

### Live/dead blue stain treatment

Lyophilised vials of Live/Dead Fixable Blue viability stain (Invitrogen) were resuspended at 1× concentration in 50 μL of room temperature (RT) DMSO, as per the manufacturer's instructions. Stock aliquots of 4 μL stored at −80°C for later use and were found to have stable staining intensity for at least five freeze/thaw cycles. When required, an aliquot was thawed and diluted in PBS to a working concentration of 1/40 for final use at 1.25 μL per 50 μL total stain volume (1/1600 final dilution).

### Spectral flow cytometry staining procedure and sample acquisition

Single vials of 1 mL PBMC were thawed in a 37°C water bath and diluted with 10 mL T‐cell medium (RPMI‐1640 supplemented with 0.3 g L^−1^ L‐glutamine (Sigma‐Aldrich), 10% FCS, 1% penicillin–streptomycin (Thermo Fisher) and 2‐Mercaptoethanol (Sigma‐Aldrich)) before centrifugation at 820 *g* for 10 min at RT. All remaining centrifugations were performed at 500 *g* for 5 min at RT. After washing again in T‐cell media, samples were strained through a 70‐μm filter and cell count and viability were determined using an automated cell counter (Nexcelom, Lawrence, MA, USA) before splitting into fractions that were subjected to staining with the resting panel or overnight stimulation for later staining with the activated panel (see below). The total staining volume for all stains was 50 μL.

*Resting panel*: Two million cells were washed in PBS and stained with Live/Dead Blue Fixable Viability Stain in PBS for 20 min at RT. After washing with FACS wash (0.1% sodium azide and 0.2% bovine serum albumin in PBS), cells were surface‐stained for 20 min at room temperature with an antibody cocktail in Brilliant Stain buffer (BD Biosciences) (Table 1, Supplementary table 1). Cells were washed and resuspended in 500 μL FoxP3 fixation/permeabilisation solution (eBiosciences, San Diego, CA, USA) for 45 min at room temperature on an orbital shaker in the dark. After two washes with permeabilisation buffer (eBiosciences), cells were stained for intracellular markers for 40 min at room temperature in the presence of permeabilisation buffer and Brilliant stain buffer (BD Biosciences). Cells were washed twice with permeabilisation buffer, resuspended in FACS wash and stored at 4°C in the dark overnight until acquisition.

*Activated panel*: 96‐well U‐bottom plates were coated with 1 μg mL^−1^ CD3 (OKT3, eBioscience) for 2 h at 37°C and then washed three times with PBS. 1.5 million cells were added and incubated for 2 h in T‐cell medium containing 5 μg mL^−1^ CD28 (CD28.2, eBioscience) before overnight incubation in the presence of 3 μg mL^−1^ Brefeldin A and 2 μM Monensin (eBioscience). An unstimulated control (incubation without CD3 or CD28) was also included. The following day, cells were washed and stained as per the resting panel protocol (Table 1, Supplementary table 1).

Both panels were acquired on a 5‐laser Cytek Aurora using standard Cytek Assay Settings adjusted daily using SpectroFlo Quality Control (QC) beads (Cytek Biosciences, Fremont, CA, USA) as per the manufacturer's recommendations. FSC and SSC were adjusted to optimally identify the lymphocyte population, and the FSC area scaling factor was set to 0.95. Samples were acquired using the live unmixing functionality and run at a medium flow rate, averaging 3000–5000 events/s.

### Spectral flow data quality control and manual analysis

All samples were analysed using FlowJo v10.8 (BD Life Sciences), with minor compensation adjustments performed as required after fluorochrome‐specific biexponential scaling. Only samples with high viability (> 80%) and stability (assessed using CD3 BUV805 *versus* time) were included in this study. Manual identification of T‐cell populations was performed as described previously (Figure 1, Supplementary table 2),^53^ except that for the activated panel, T and NK cells were gated together (CD19^−^ CD3^+/−^) due to variable CD3 staining after CD3/28 stimulation, and CD4^+^ and CD8^+^ T cells were gated as TCRγδ^−^ CD4^+^ and TCRγδ^−^ CD8^hi^, respectively (NK cells are predominantly CD4^−^ CD8^−/lo^) (Figure 2, Supplementary table 3). Additional markers for phenotypic characterisation of subsets were typically gated on bulk CD4^+^ or CD8^+^ populations before application to derivative memory subsets. Select markers, such as Inducible T‐cell Co‐Stimulator (ICOS), T‐cell Immunoglobulin and Mucin‐domain‐containing‐3 (TIM‐3) and PD‐1, were gated on populations where their expression was most well‐defined (ICOS on γδT cells, TIM‐3 on Tcm and PD‐1 on TemRO cells) (Supplementary figures 3 and 4). Data were analysed using GraphPad Prism v8.3.1 and the tidyverse v1.3.1^56^ and ggpubr v0.4.0^57^ packages in the R programming environment v4.1.3.^58^

### High dimensional analysis and normalisation

Dimensionality reduction was performed on downsampled, concatenated lymphocyte events from all patient and control samples (10 000 events per sample; debris, doublets, potential erythrocyte contamination^59^ and dead cells removed) using the UMAP FlowJo plugin^60^ with the following settings: metric = Euclidean, nearest neighbours = 30, minimum distance = 0.5, components = 2; all markers except those used for pre‐gating of live lymphocytes (CD45 and Live/Dead Blue Viability). Following UMAP analysis, X‐Shift clustering^61^ was performed on manually gated T cells (resting panel) or T + NK cells (activated panel) using T‐cell population markers (TCRγδ, CD4, CD8, CD25, CD45RA, CD127, CCR7 and CXCR5 for the resting panel) with the following settings: metric = Euclidean, nearest neighbours = 132 (resting panel) or 153 (activated panel) and subsampling limit = 100 000. Clusters were identified manually and subsequently analysed using the FlowJo ClusterExplorer plugin to generate marker expression heatmaps and differential abundances in the control and patient groups. Post‐acquisition data normalisation was performed using CytoNorm^41^ through the FlowJo plugin with default settings (quartiles = 101, clusters = 10).

### Statistical analysis

All statistical analyses of populations from healthy controls and patients were performed using the unpaired non‐parametric Mann–Whitney test; values < 0.05 were considered significant. In figures, *P*‐values are assigned as follows: *P < 0.05, **P < 0.01, ***P < 0.001. Multiple comparison correction using the Bonferroni method was performed for major T‐cell lineages (γδT, CD4, CD8; adjusted *P*‐value cut‐off 0.016) and for the 18 markers analysed (adjusted *P*‐value cut‐off 0.0028).

## Author contributions

**Jack M Edwards:** Conceptualization; data curation; formal analysis; investigation; methodology; project administration; software; visualization; writing – original draft; writing – review and editing. **Miles C Andrews:** Data curation; project administration; resources; supervision; writing – original draft; writing – review and editing. **Hayley Burridge:** Project administration; resources. **Robin Smith:** Project administration; resources. **Carole Owens:** Project administration; resources. **Mark Edinger:** Methodology. **Katherine Pilkington:** Methodology. **Juliette Desfrancois:** Methodology. **Mark Shackleton:** Funding acquisition; resources. **Sashendra Senthi:** Conceptualization; funding acquisition; investigation; methodology; project administration; resources; supervision; visualization; writing – original draft; writing – review and editing. **Menno C van Zelm:** Conceptualization; investigation; methodology; project administration; resources; supervision; visualization; writing – original draft; writing – review and editing.

## Conflict of interest

MCA reports advisory board participation for MSD Australia, honoraria from MSD Australia and Pierre Fabre Australia, and research funds to institutions from MSD Australia and BMS Australia, all unrelated to the current work. KP, ME and JD are employees of Cytek Biosciences, Inc., the manufacturer of the Aurora full‐spectrum flow cytometer used in this study.

## Supporting information


Supplementary table 1
Supplementary table 2
Supplementary table 3
Supplementary table 4
Supplementary table 5
Supplementary figure 1
Supplementary figure 2
Supplementary figure 3
Supplementary figure 4
Supplementary figure 5
Supplementary figure 6
Supplementary figure 7
Supplementary figure 8
Supplementary figure 9

## Data Availability

Flow cytometry files are available from FlowRepository (Experiment ID: FR‐FCM‐Z6G4). Further data are available upon reasonable request.
