# Supplementary material for: Design, optimisation and standardisation of a high‐dimensional spectral flow cytometry workflow assessing T‐cell immunophenotype in patients with melanoma
Source: Clin Transl Immunology. 2023 Sep 7;12(9):e1466. doi: 10.1002/cti2.1466 (PMC10484688; doi:10.1002/cti2.1466)
Supplement: Supplementary file 1 — Supplementary table 1 Supplementary table 2 Supplementary table 3 Supplementary table 4 Supplementary table 5 Supplementary figure 1 Supplementary figure 2 Supplementary figure 3 Supplementary figure 4 Supplementary figure 5 Supplementary figure 6 Supplementary figure 7 Supplementary figure 8 Supplementary figure 9 [file CTI2-12-e1466-s001.pdf]

Supplementary table 1: Spectral panel 1 (resting) and panel 2 (activated) reagents.

Target	Stain	Fluorochrome	Clone	Supplier	RRID^	Test use	
						µL/test	µg mL^{-1}
CD3	Surface	BUV805	UCHT1	BD Biosciences	AB_2800945	1.25	2.5
CD4	Surface	cFluor YG584	SK3	Cytek	AB_2870183	1.25	0.5
CD8	Surface	Spark Blue 550	SK1	Biolegend	AB_2885083	0.25	0.1
CD16	Surface	BV510	3GA	BD Biosciences	AB_2819982	1.25	5
CD19	Surface	BUV563	SJ25C1	BD Biosciences	AB_2744296	0.3	0.6
CD25	Surface	PE-Fire700	M-A251	Biolegend	AB_2870202	2.5	2.5
CD39	Surface	APC-Fire750	A1	Biolegend	AB_2876678	0.5	0.5
CD45	Surface	PerCP	HI30	Biolegend	AB_2650838	0.5	2
CD45RA	Surface	BUV496	HI100	BD Biosciences	AB_893341	0.04	0.0064
CD56	Surface	BUV737	B159	BD Biosciences	AB_2874456	0.4	1.6
CD57	Surface	PB	HNK-1	Biolegend	AB_2871176	0.2	0.2
CD95	Surface	PE-Dazzle594	DX2	Biolegend	AB_2562458	0.625	1.25
CD127	Surface	R718	HIL-7R-M21	BD Biosciences	AB_2564221	2.5	2.5
CCR7	Surface	BV750	GO43H7	Biolegend	AB_2869977	2.5	10
CTLA-4	Intracell.	BV785	BNI3	Biolegend	AB_2810582	0.6	1.2
CXCR5	Surface	BV605	RF8B2	BD Biosciences	AB_2740110	2.5	10
EOMES	Intracell.	PE-Cy7	WD1928	ThermoFisher	AB_2573456	2.5	0.6
GrzmB	Intracell.	BV510	GB11	BD Biosciences	AB_2738174	0.3	1.2
ICOS	Surface	BUV661	DX29	BD Biosciences	AB_2871056	1.25	5
IFNγ	Intracell.	BV605	B27	BD Biosciences	AB_2737926	0.6	1.2
IL-2	Intracell.	AF488	MQ1-17H12	BD Biosciences	AB_2738566	2.5	2.5
IL-4	Intracell.	APC	MP4-25D2	Biolegend	AB_2295923	2.5	0.2
IL-10	Intracell.	BV421	JES3-9D7	BD Biosciences	AB_493368	2.5	2.5
IL-17A	Intracell.	PE-Cy7	BL168	Biolegend	AB_315131	0.2	0.6
IRF4	Intracell.	AF647	IRF4.3E4	Biolegend	AB_2564047	1.25	2.5
Ki67	Intracell.	BUV395	B59	BD Biosciences	AB_2738577	1.25	7.5
KLRG1	Surface	APC	2F1	Biolegend	AB_10645509	0.3	0.3
PD-1	Surface	BV786	EH12.1	BD Biosciences	AB_2738425	1.25	5
Tbet	Intracell.	PE-Cy5	4B10	ThermoFisher	AB_2815071	0.4	1.6
TCRγδ	Surface	PerCP-Vio700	REA591	Miltenyi	AB_2733074	1	8
TIGIT	Surface	BV421	A5153G	Biolegend	AB_2632925	2.5	2.5
TIM-3	Surface	BB515	7D3	BD Biosciences	AB_2744368	2.5	5
TNFα	Intracell.	BUV395	MAb11	BD Biosciences	AB_2738533	0.5	2
TOX	Intracell.	PE	TXRX10	ThermoFisher	AB_10855034	0.625	2.5
Viability	Surface	Live/Dead Blue	L34962	ThermoFisher	*NA*	1.25	*NA*

^RRID: Research resource identifier

Supplementary table 2: Panel 1 (resting) population definitions.

	**Population name**	**Phenotype definition**
	All populations gated from live lymphocytes:	CD45^{+} SSC^{low} Live/Dead Blue^{-}
1	B cells	CD3^{-} CD19^{+}
2	NK cells	CD3^{-} CD19^{-} CD16/CD56^{+}
3	T cells	CD3^{+} CD19^{-}
4	∟TCRγδ^{+}	CD3^{+} CD19^{-} TCRγδ^{+}
7	∟TCRαβ^{+}	CD3^{+} CD19^{-} TCRγδ^{-}
10	∟CD4^{-} CD8^{-}	CD3^{+} CD19^{-} TCRγδ^{-} CD4^{-} CD8^{-}
11	∟CD4 T cells	CD3^{+} CD19^{-} TCRγδ^{-} CD4^{+} CD8^{-}
12	∟Treg	CD3^{+} CD19^{-} TCRγδ^{-} CD4^{+} CD25^{+} CD127$^{-/lo}$
13	∟NOT Treg	CD3^{+} CD19^{-} TCRγδ^{-} CD4^{+} CD25$^{+/lo}$ CD127$^{+/-}$
14	∟Naive/stem-like	CD3^{+} CD19^{-} TCRγδ^{-} CD4^{+} CD25$^{+/lo}$ CD127$^{+/-}$ CD45RA^{+} CCR7^{+}
15	∟Tnaive	CD3^{+} CD19^{-} TCRγδ^{-} CD4^{+} CD25$^{+/lo}$ CD127$^{+/-}$ CD45RA^{+} CCR7^{+} CD95^{-}
16	∟Tscm	CD3^{+} CD19^{-} TCRγδ^{-} CD4^{+} CD25$^{+/lo}$ CD127$^{+/-}$ CD45RA^{+} CCR7^{+} CD95^{+}
17	∟Tcm	CD3^{+} CD19^{-} TCRγδ^{-} CD4^{+} CD25$^{+/lo}$ CD127$^{+/-}$ CD45RA^{-} CCR7^{+}
18	∟TemRO	CD3^{+} CD19^{-} TCRγδ^{-} CD4^{+} CD25$^{+/lo}$ CD127$^{+/-}$ CD45RA^{-} CCR7^{-}
19	∟TemRA	CD3^{+} CD19^{-} TCRγδ^{-} CD4^{+} CD25$^{+/lo}$ CD127$^{+/-}$ CD45RA^{+} CCR7^{-}
20	∟Tfh	CD3^{+} CD19^{-} TCRγδ^{-} CD4^{+} CD25$^{+/lo}$ CD127$^{+/-}$ CD45RA^{+} CXCR5^{+}
21	∟CD8 T cells	CD3^{+} CD19^{-} TCRγδ^{-} CD8^{+}
22	∟Naive/stem-like	CD3^{+} CD19^{-} TCRγδ^{-} CD8^{+} CD45RA^{+} CCR7^{+}
23	∟Tnaive	CD3^{+} CD19^{-} TCRγδ^{-} CD4^{+} CD25$^{+/lo}$ CD127$^{+/-}$ CD45RA^{+} CCR7^{+} CD95^{-}
24	∟Tscm	CD3^{+} CD19^{-} TCRγδ^{-} CD4^{+} CD25$^{+/lo}$ CD127$^{+/-}$ CD45RA^{+} CCR7^{+} CD95^{+}
25	∟Tcm	CD3^{+} CD19^{-} TCRγδ^{-} CD8^{+} CD45RA^{-} CCR7^{+}
26	∟TemRO	CD3^{+} CD19^{-} TCRγδ^{-} CD8^{+} CD45RA^{-} CCR7^{-}
27	∟TemRA	CD3^{+} CD19^{-} TCRγδ^{-} CD8^{+} CD45RA^{+} CCR7^{-}

Supplementary table 3: Panel 2 (activated) population definitions.

	Population name	Phenotype definition
	All populations gated from live lymphocytes:	CD45^{+} SSC^{low} Live/Dead Blue^{-}
1	T + NK cells	CD19^{-}
2	∟ TCRγδ^{+}	CD19^{-} TCRγδ^{+}
3	∟ TCRγδ^{-}	CD19^{-} TCRγδ^{-}
4	∟ CD4^{+} T cells	CD19^{-} TCRγδ^{-} CD4^{+}
5	∟ Treg	CD19^{-} TCRγδ^{-} CD4^{+} CD25^{+} CD127$^{-/lo}$
6	∟ NOT Treg	CD19^{-} TCRγδ^{-} CD4^{+} CD25$^{+/lo}$ CD127$^{+/-}$
7	∟ Naive/stem-like	CD3^{+} CD19^{-} TCRγδ^{-} CD4^{+} CD25$^{+/lo}$ CD127$^{+/-}$ CD45RA^{+} CCR7^{+}
8	∟ Tnaive	CD3^{+} CD19^{-} TCRγδ^{-} CD4^{+} CD25$^{+/lo}$ CD127$^{+/-}$ CD45RA^{+} CCR7^{+} CD95^{-}
9	∟ Tscm	CD3^{+} CD19^{-} TCRγδ^{-} CD4^{+} CD25$^{+/lo}$ CD127$^{+/-}$ CD45RA^{+} CCR7^{+} CD95^{+}
10	∟ Tcm	CD19^{-} TCRγδ^{-} CD4^{+} CD25$^{+/lo}$ CD127$^{+/-}$ CD45RA^{-} CCR7^{+}
11	∟ TemRO	CD19^{-} TCRγδ^{-} CD4^{+} CD25$^{+/lo}$ CD127$^{+/-}$ CD45RA^{-} CCR7^{-}
12	∟ TemRA	CD19^{-} TCRγδ^{-} CD4^{+} CD25$^{+/lo}$ CD127$^{+/-}$ CD45RA^{+} CCR7^{-}
13	∟ Th1	CD19^{-} TCRγδ^{-} CD4^{+} CD25$^{+/lo}$ CD127$^{+/-}$ IL-17A^{-} IFNγ^{+}
14	∟ Th2	CD19^{-} TCRγδ^{-} CD4^{+} CD25$^{+/lo}$ CD127$^{+/-}$ IL-17A^{-} IFNγ^{-} IL-4^{+}
15	∟ Th17	CD19^{-} TCRγδ^{-} CD4^{+} CD25$^{+/lo}$ CD127$^{+/-}$ IL-17A^{+} IFNγ^{-}
16	∟ CD8^{+} T cells	CD19^{-} TCRγδ^{-} CD8^{+}
17	∟ Naive/stem-like	CD3^{+} CD19^{-} TCRγδ^{-} CD4^{+} CD25$^{+/lo}$ CD127$^{+/-}$ CD45RA^{+} CCR7^{+}
18	∟ Tnaive	CD3^{+} CD19^{-} TCRγδ^{-} CD4^{+} CD25$^{+/lo}$ CD127$^{+/-}$ CD45RA^{+} CCR7^{+} CD95^{-}
19	∟ Tscm	CD3^{+} CD19^{-} TCRγδ^{-} CD4^{+} CD25$^{+/lo}$ CD127$^{+/-}$ CD45RA^{+} CCR7^{+} CD95^{+}
20	∟ Tcm	CD19^{-} TCRγδ^{-} CD8^{+} CD45RA^{-} CCR7^{-}
21	∟ TemRO	CD19^{-} TCRγδ^{-} CD8^{+} CD45RA^{-} CCR7^{-}
22	∟ TemRA	CD19^{-} TCRγδ^{-} CD8^{+} CD45RA^{+} CCR7^{-}

Supplementary table 4: TruCount antibody panel.

					Test use	
**Target**	**Fluorochrome**	**Clone**	**Vendor**	**RRID^**	**µL/test**	**µg mL^{-1}**
CD3	FITC	UCHT1	BD Biosciences	AB_395739	3	0.56
CD4	BV605	RPA-T4	BD Biosciences	AB_2744420	0.5	0.36
CD8	BV510	SKI	BD Biosciences	AB_2722546	0.5	1.43
CD14	APC-H7	MφP9	BD Biosciences	AB_1645725	1	0.36
CD16	PE	3GA	Biolegend	AB_314208	0.7	1.00
CD19	PE-Cy7	SJ25C1	BD Biosciences	AB_396893	1	0.71
CD45	PerCP-Cy5.5	2D1	BD Biosciences	AB_400194	4	0.34
CD56	PE	B159	BD Biosciences	AB_395906	5	1.79
HLA-DR	APC	L243	Biolegend	AB_314688	2	0.71

^RRID: Research resource identifier

Supplementary table 5: TruCount panel population definitions.

	Population name	Phenotype definition
1	Granulocytes	SSC^{high} $CD45^{+}$
2	∟neutrophils	SSC^{high} $CD45^{+}$ $CD16/56^{+}$
3	∟eosinophils	SSC^{high} $CD45^{+}$ $CD16/56^{-}$
	Mononuclear cells	SSC^{inter} $CD45^{+}$
4	∟M-MDSCs	SSC^{inter} $CD45^{+}$ $CD3^{-}$ $CD19^{-}$ $HLA\text{-}DR^{-}$ $CD16/56^{-}$ $CD14^{+}$
5	∟M-DCs	SSC^{inter} $CD45^{+}$ $CD3^{-}$ $CD19^{-}$ $HLA\text{-}DR^{+}$ $CD16/56^{-}$ $CD14^{-}$
6	∟Monocytes	SSC^{inter} $CD45^{+}$ $CD3^{-}$ $CD19^{-}$ $HLA\text{-}DR^{+}$ $CD16/56^{+/-}$ $CD14^{+/-}$
7	∟classical	SSC^{inter} $CD45^{+}$ $CD3^{-}$ $CD19^{-}$ $HLA\text{-}DR^{+}$ $CD16/56^{-}$ $CD14^{+}$
8	∟intermediate	SSC^{inter} $CD45^{+}$ $CD3^{-}$ $CD19^{-}$ $HLA\text{-}DR^{+}$ $CD16/56^{+}$ $CD14^{+/-}$
9	∟non-classical	SSC^{inter} $CD45^{+}$ $CD3^{-}$ $CD19^{-}$ $HLA\text{-}DR^{+}$ $CD16/56^{+}$ $CD14^{-}$
10	Lymphocytes	SSC^{low} $CD45^{+}$
11	∟B cells	SSC^{low} $CD45^{+}$ $CD3^{-}$ $CD19^{+}$
12	∟NK cells	SSC^{low} $CD45^{+}$ $CD3^{-}$ $CD16/56^{+}$
13	∟T cells	SSC^{low} $CD45^{+}$ $CD19^{-}$ $CD3^{+}$
14	∟$CD4^{+}$ T cells	SSC^{low} $CD45^{+}$ $CD19^{-}$ $CD3^{+}$ $CD4^{+}$
15	∟$CD8^{+}$ T cells	SSC^{low} $CD45^{+}$ $CD19^{-}$ $CD3^{+}$ $CD8^{+}$

a

Similarity: 0 — 0.5 — 1

Panel 1	BUV395	LIVE DEAD Blue	BUV496	BUV563	BUV661	BUV737	BUV805	BV421	Pacific Blue	BV510	BV605	BV750	BV786	BB515	Spark Blue 550	PerCP	PerCP-Vio700	PE	cFluor YG584	PE-Dazzle594	PE-Cy5	PE-Fire 700	PE-Cy7	APC	Alexa Fluor 647	R718	APC-Fire 750
BUV395	1																										
LIVE DEAD Blue	0.51	1																									
BUV496	0.25	0.58	1																								
BUV563	0.05	0.09	0.29	1																							
BUV661	0.04	0.03	0.02	0.06	1																						
BUV737	0.03	0.02	0.01	0.01	0.37	1																					
BUV805	0.11	0.06	0.03	0.01	0.1	0.39	1																				
BV421	0.05	0.26	0.09	0.01	0	0	0.01	1																			
Pacific Blue	0.01	0.21	0.13	0.01	0	0	0	0.78	1																		
BV510	0.02	0.2	0.48	0.22	0.04	0.02	0	0.17	0.36	1																	
BV605	0.01	0.02	0.05	0.2	0.17	0.05	0.01	0.06	0.06	0.4	1																
BV750	0	0.01	0	0	0.09	0.37	0.14	0.05	0.04	0.04	0.1	1															
BV786	0	0.04	0.01	0	0.07	0.29	0.23	0.2	0.15	0.06	0.09	0.86	1														
BB515	0.01	0.01	0.07	0.04	0	0	0	0	0	0.01	0	0	0	1													
Spark Blue 550	0.02	0.01	0.09	0.15	0.01	0.01	0	0.02	0.05	0.3	0.16	0.01	0.02	0.53	1												
PerCP	0	0.01	0.01	0.04	0.38	0.15	0.03	0.01	0.02	0.01	0.18	0.13	0.08	0.01	0.06	1											
PerCP-Vio700	0	0	0	0.02	0.3	0.37	0.07	0	0	0.02	0.16	0.39	0.3	0	0.06	0.58	1										
PE	0.01	0	0.03	0.51	0.03	0	0	0	0.01	0.12	0.28	0	0	0.05	0.24	0.06	0.04	1									
cFluor YG584	0	0	0.01	0.46	0.06	0	0	0	0	0.04	0.3	0	0	0.01	0.05	0.07	0.05	0.88	1								
PE-Dazzle594	0.01	0	0.02	0.36	0.13	0.02	0	0	0.01	0.09	0.47	0.01	0.01	0.03	0.19	0.27	0.19	0.67	0.72	1							
PE-Cy5	0	0	0	0.07	0.42	0.1	0.01	0	0	0.01	0.21	0.05	0.03	0.01	0.06	0.77	0.55	0.14	0.19	0.43	1						
PE-Fire 700	0	0	0	0.04	0.28	0.19	0.03	0	0	0.01	0.12	0.09	0.07	0	0.04	0.47	0.78	0.07	0.09	0.25	0.66	1					
PE-Cy7	0	0	0	0.01	0.05	0.14	0.08	0	0	0	0.03	0.15	0.17	0	0.01	0.09	0.31	0.02	0.03	0.05	0.14	0.31	1				
APC	0	0.01	0	0.03	0.78	0.24	0.02	0	0	0.02	0.14	0.07	0.04	0	0.01	0.34	0.3	0.04	0.08	0.16	0.52	0.3	0.05	1			
Alexa Fluor 647	0	0.01	0	0.01	0.71	0.22	0.02	0	0	0	0.03	0.02	0.01	0	0	0.23	0.23	0.01	0.03	0.06	0.32	0.23	0.03	0.9	1		
R718	0	0	0	0	0.41	0.52	0.08	0	0	0	0.02	0.12	0.09	0.01	0.01	0.1	0.34	0	0.01	0.02	0.16	0.27	0.08	0.47	0.52	1	
APC-Fire 750	0	0	0.01	0	0.13	0.31	0.21	0	0	0.01	0.02	0.2	0.23	0	0	0.04	0.19	0	0	0.01	0.07	0.14	0.32	0.16	0.16	0.46	1

Complexity Index: 10.72

b

Panel 2	BUV395	LIVE DEAD Blue	BUV496	BUV563	BUV805	BV421	BV510	BV605	BV750	BV786	Alexa Fluor 488	Spark Blue 550	PerCP	PerCP-Vio700	cFluor YG584	PE-Dazzle594	PE-Fire 700	PE-Cy7	APC	R718
BUV395	1																			
LIVE DEAD Blue	0.51	1																		
BUV496	0.25	0.58	1																	
BUV563	0.05	0.09	0.29	1																
BUV805	0.11	0.06	0.03	0.01	1															
BV421	0.05	0.26	0.09	0.01	0.01	1														
BV510	0.02	0.2	0.48	0.22	0	0.17	1													
BV605	0.01	0.02	0.05	0.2	0.01	0.06	0.4	1												
BV750	0	0.01	0	0	0.14	0.05	0.04	0.1	1											
BV786	0	0.04	0.01	0	0.23	0.2	0.06	0.09	0.86	1										
Alexa Fluor 488	0.01	0	0.06	0.05	0	0	0.01	0	0	0	1									
Spark Blue 550	0.02	0.01	0.09	0.15	0	0.02	0.3	0.16	0.01	0.02	0.63	1								
PerCP	0	0.01	0.01	0.04	0.03	0.01	0.01	0.18	0.13	0.08	0.01	0.06	1							
PerCP-Vio700	0	0	0	0.02	0.07	0	0.02	0.16	0.39	0.3	0	0.06	0.58	1						
cFluor YG584	0	0	0.01	0.46	0	0	0.04	0.3	0	0	0.01	0.05	0.07	0.05	1					
PE-Dazzle594	0.01	0	0.02	0.36	0	0	0.09	0.47	0.01	0.01	0.05	0.19	0.27	0.19	0.72	1				
PE-Fire 700	0	0	0	0.04	0.03	0	0.01	0.12	0.09	0.07	0	0.04	0.47	0.78	0.09	0.25	1			
PE-Cy7	0	0	0	0.01	0.08	0	0	0.03	0.15	0.17	0	0.01	0.09	0.31	0.03	0.05	0.31	1		
APC	0	0.01	0	0.03	0.02	0	0.02	0.14	0.07	0.04	0	0.01	0.34	0.3	0.08	0.16	0.3	0.05	1	
R718	0	0	0	0	0.08	0	0	0.02	0.12	0.09	0.01	0.01	0.1	0.34	0.01	0.02	0.27	0.08	0.47	1

Complexity Index: 7.4

Supplementary figure 1: Panel complexity for **(a)** resting and **(b)** activated panels.

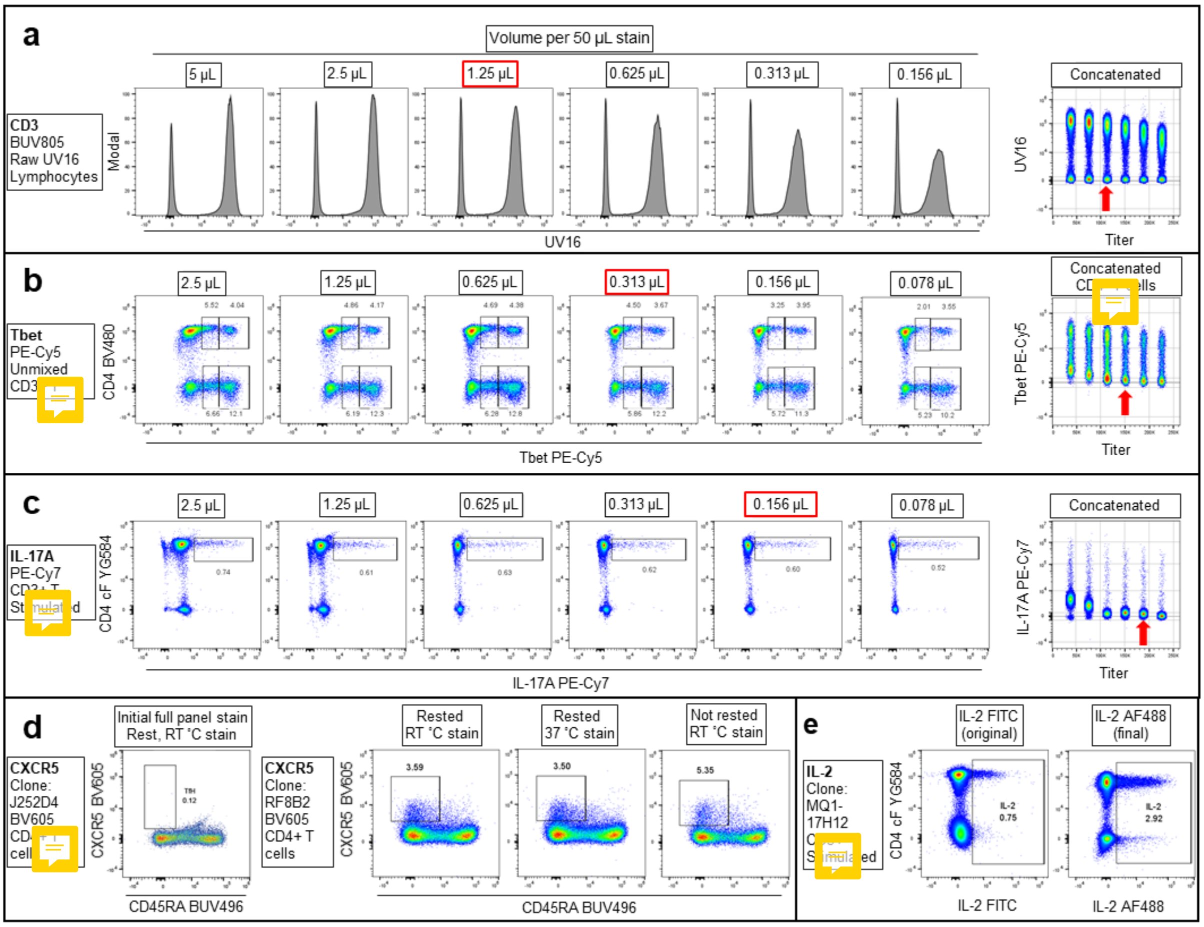

Supplementary figure 2: Panel development and optimization. All antibodies were titrated in a 6 point 2-fold dilution series. Titers are expressed as volume of antibody (µL) per 50 µL test. **(a)** Titration of CD3 BUV805. Antibodies with primary expression levels were generally titrated alone and only the peak emission channel used for analysis. The lowest titer that still gave acceptable separation was selected to limit the spreading impact of these markers. **(b)** Titration of Tbet PE-Cy5. Secondary and tertiary markers were titrated with additional markers present to enable *in situ* gating of populations of interest. Titers were selected with best separation. **(c)** Titration of IL-17A PE-Cy7. Cytokine markers were titrated after stimulation with α-CD3/CD28. **(d)** Optimization of CXCR5 and **(e)** IL-2 staining.

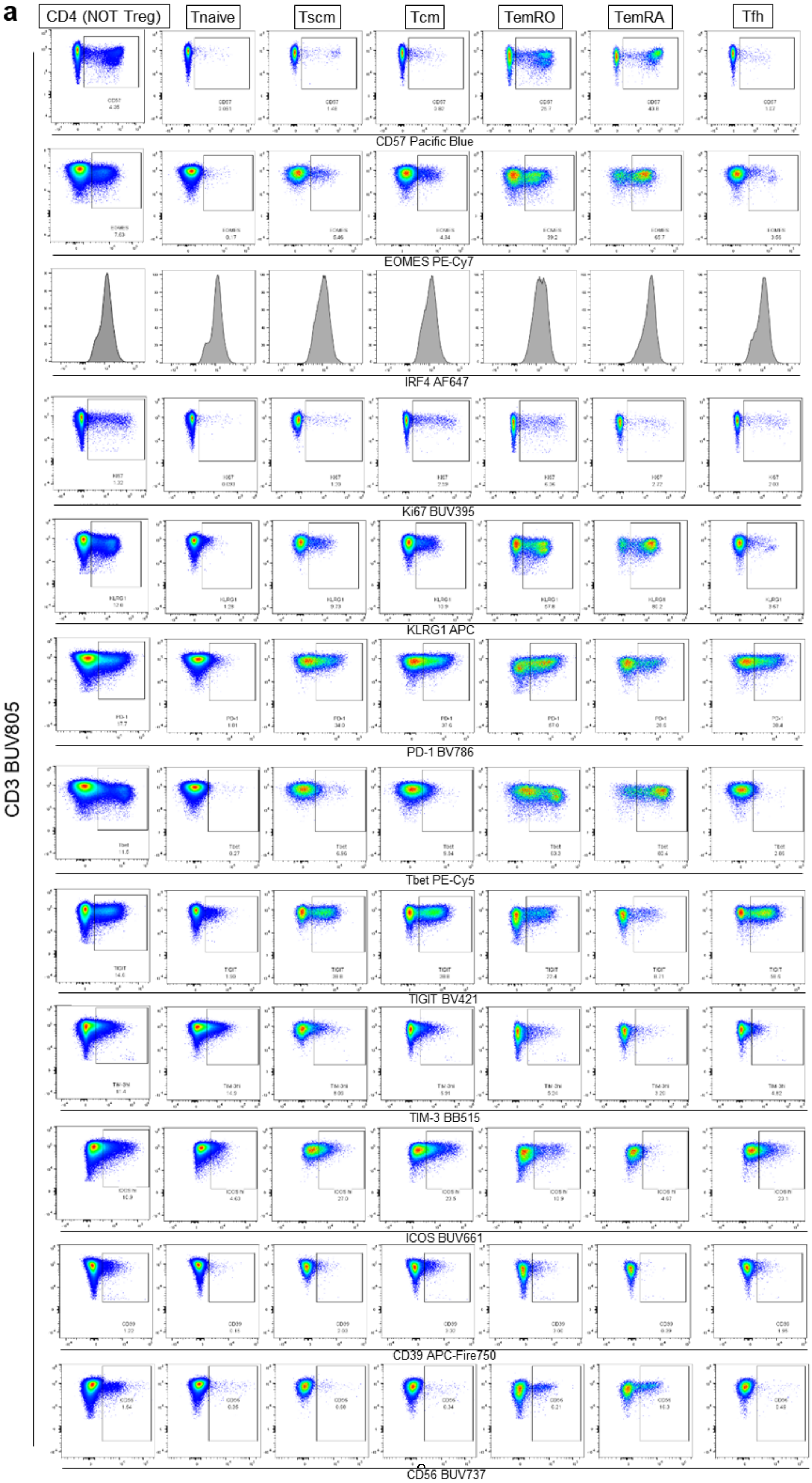
a
CD4 (NOT Treg)
Tnaive
Tscm
Tcm
TemRO
TemRA
Tfh
CD57 Pacific Blue
EOMES PE-Cy7
IRF4 AF647
Ki67 BUV395
KLRG1 APC
PD-1 BV786
Tbet PE-Cy5
TIGIT BV421
TIM-3 BB515
ICOS BUV661
CD39 APC-Fire750
CD56 BUV737
CD3 BUV805

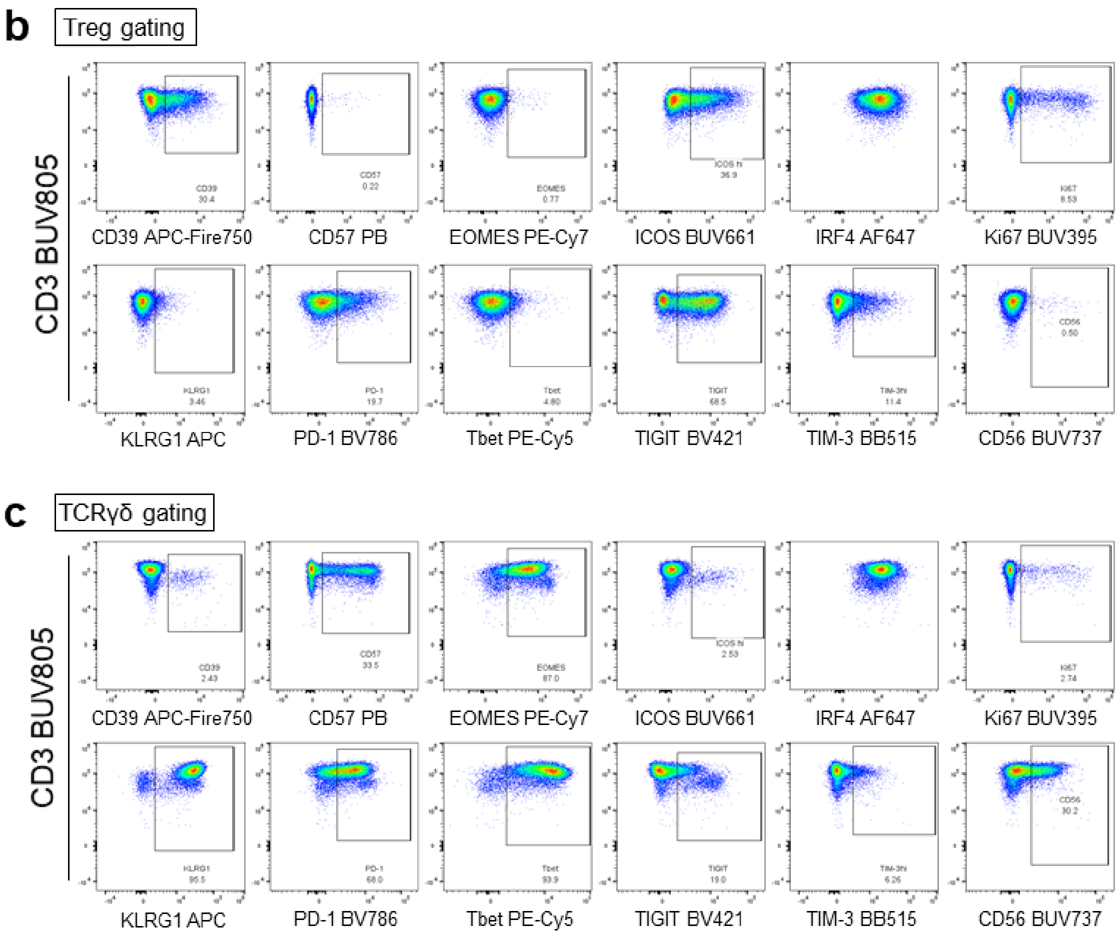

Supplementary figure 3: Example resting panel marker expression analysis for **(a)** CD4 subsets, **(b)** Tregs, and **(c)** γδT cells.

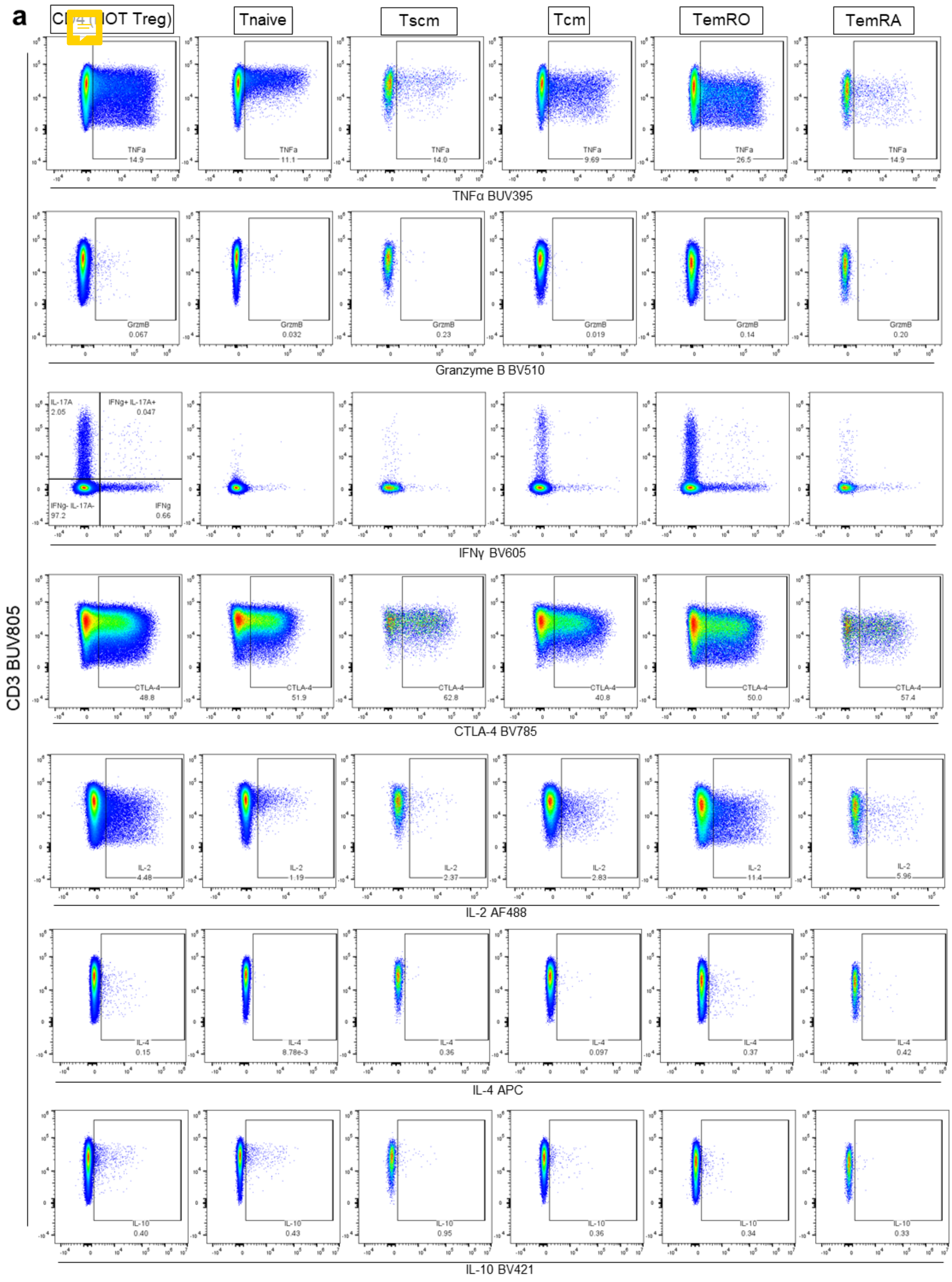
a
Tnaive
Tscm
Tcm
TemRO
TemRA
TNFα BUV395
Granzyme B BV510
IFNγ BV605
CTLA-4 BV785
IL-2 AF488
IL-4 APC
IL-10 BV421
CD3 BUV805

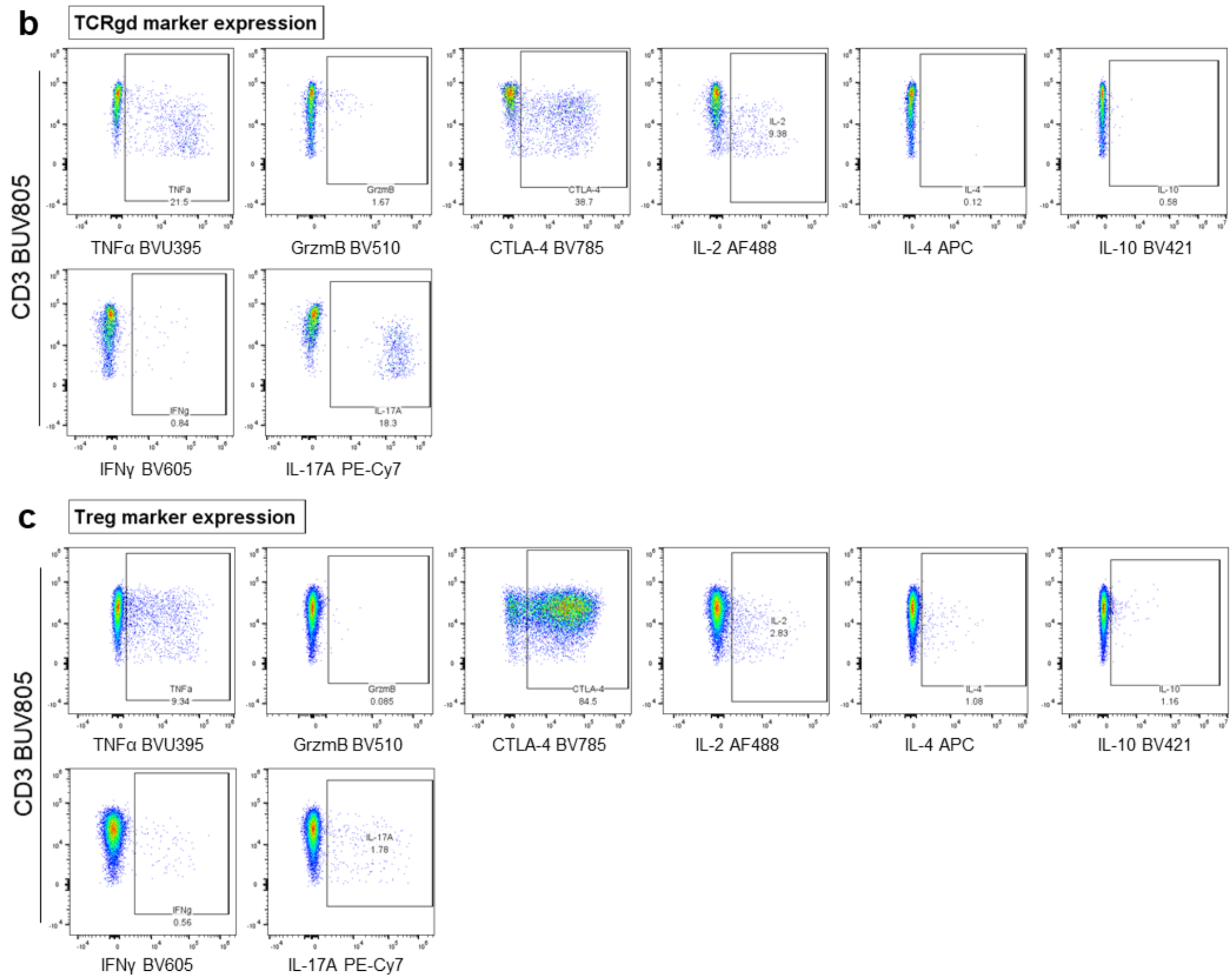

Supplementary figure 4: Example activated panel marker expression analysis for **(a)** CD4 subsets, **(b)** γδT cells, and **(c)** and Treg.

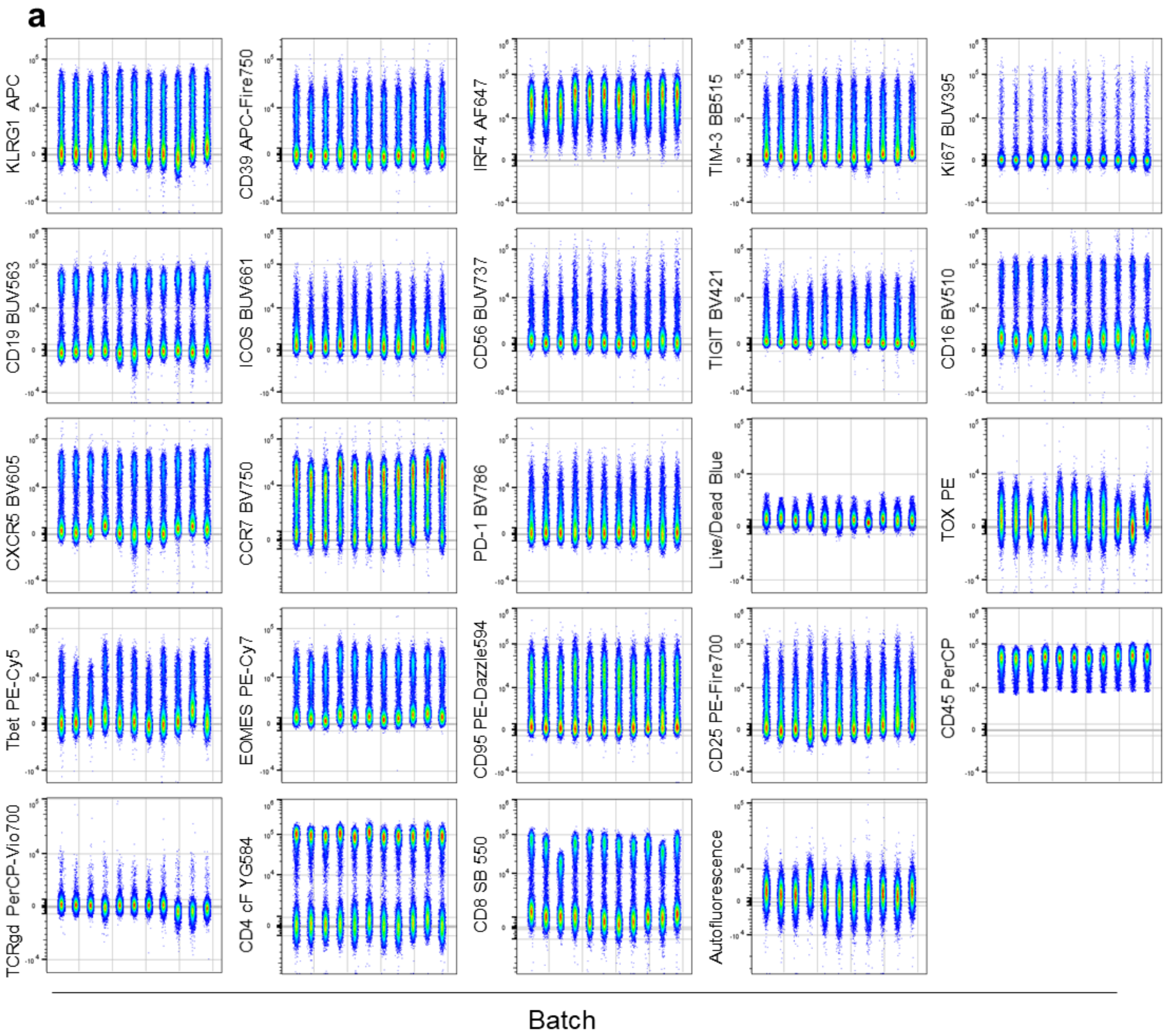

Supplementary figure 5a: Batch control sample resting panel marker expression across eleven batches. Figure panels depict live lymphocytes before normalization, and show all markers except for CD3 BUV805, CD45RA BUV496, CD57 Pacific Blue, and CD127 R718, which are shown in figure 3.

b

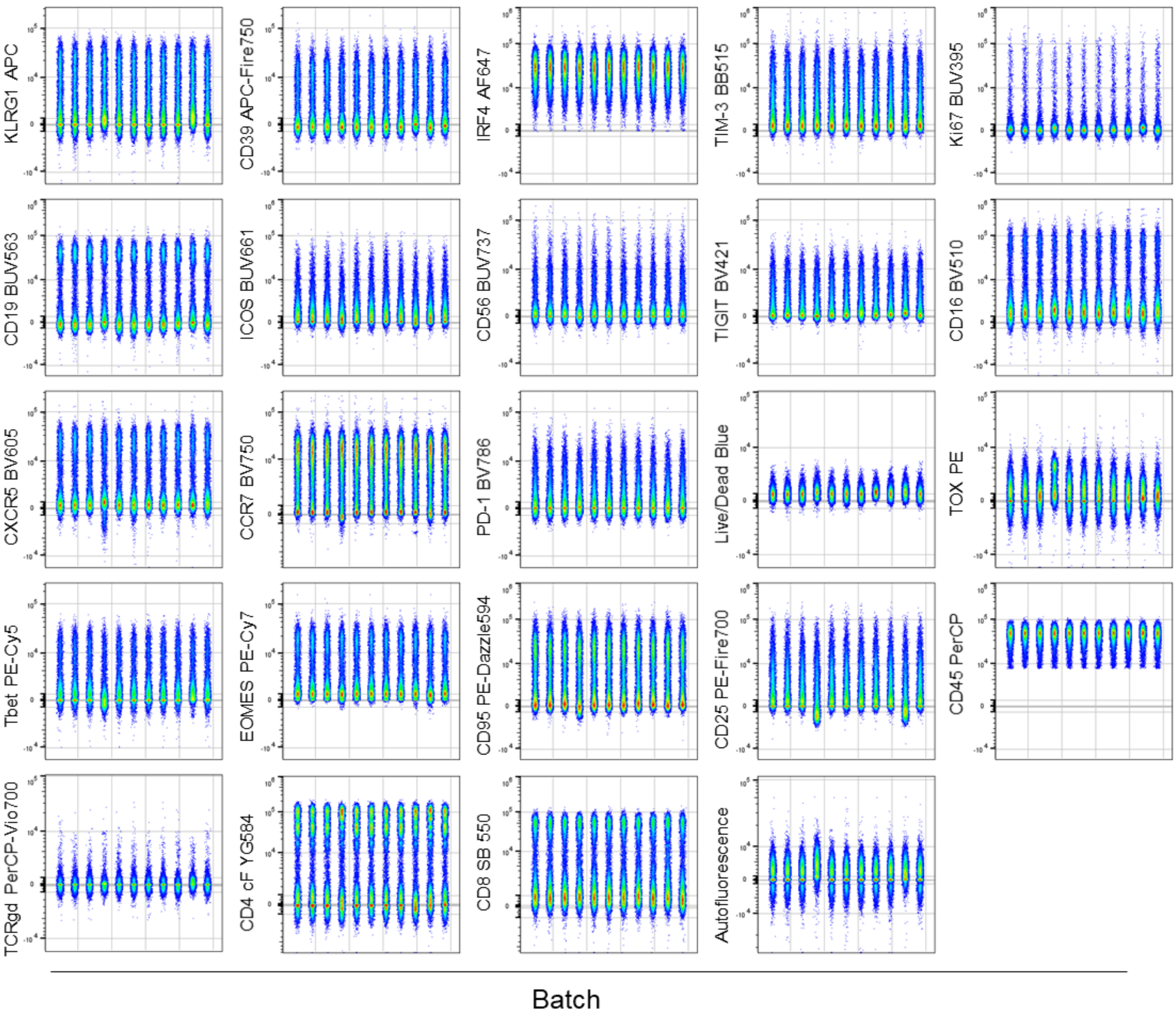

Supplementary figure 5b: Batch control sample resting panel marker expression across eleven batches. Figure panels depict live lymphocytes after normalization with CytoNorm, and show all markers except for CD3 BUV805, CD45RA BUV496, CD57 Pacific Blue, and CD127 R718, which are shown in figure 3.

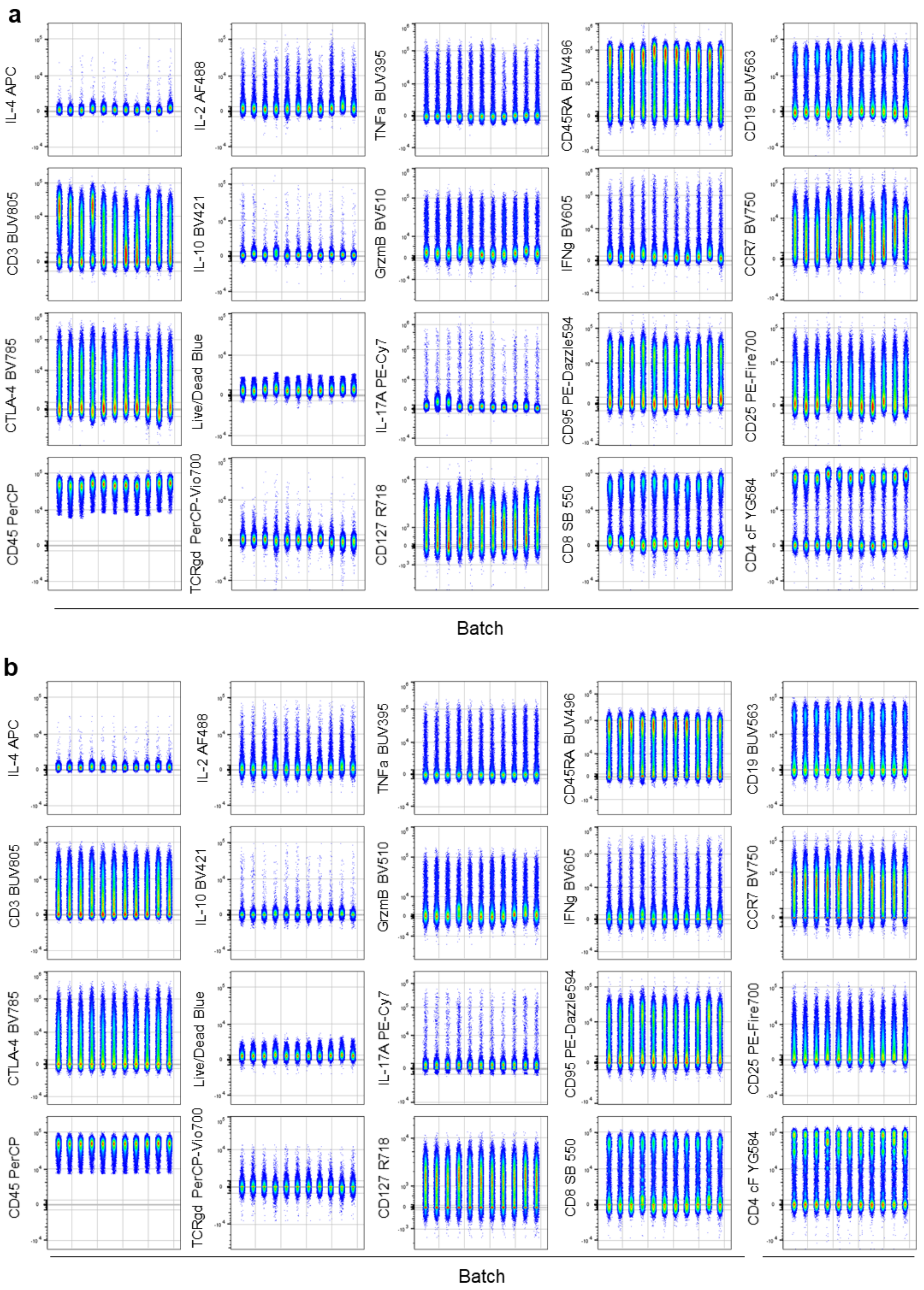

Supplementary figure 6: Batch control sample activated panel marker expression across eleven batches. Figure panels depict live lymphocytes **(A)** before and **(B)** after normalization with CytoNorm.

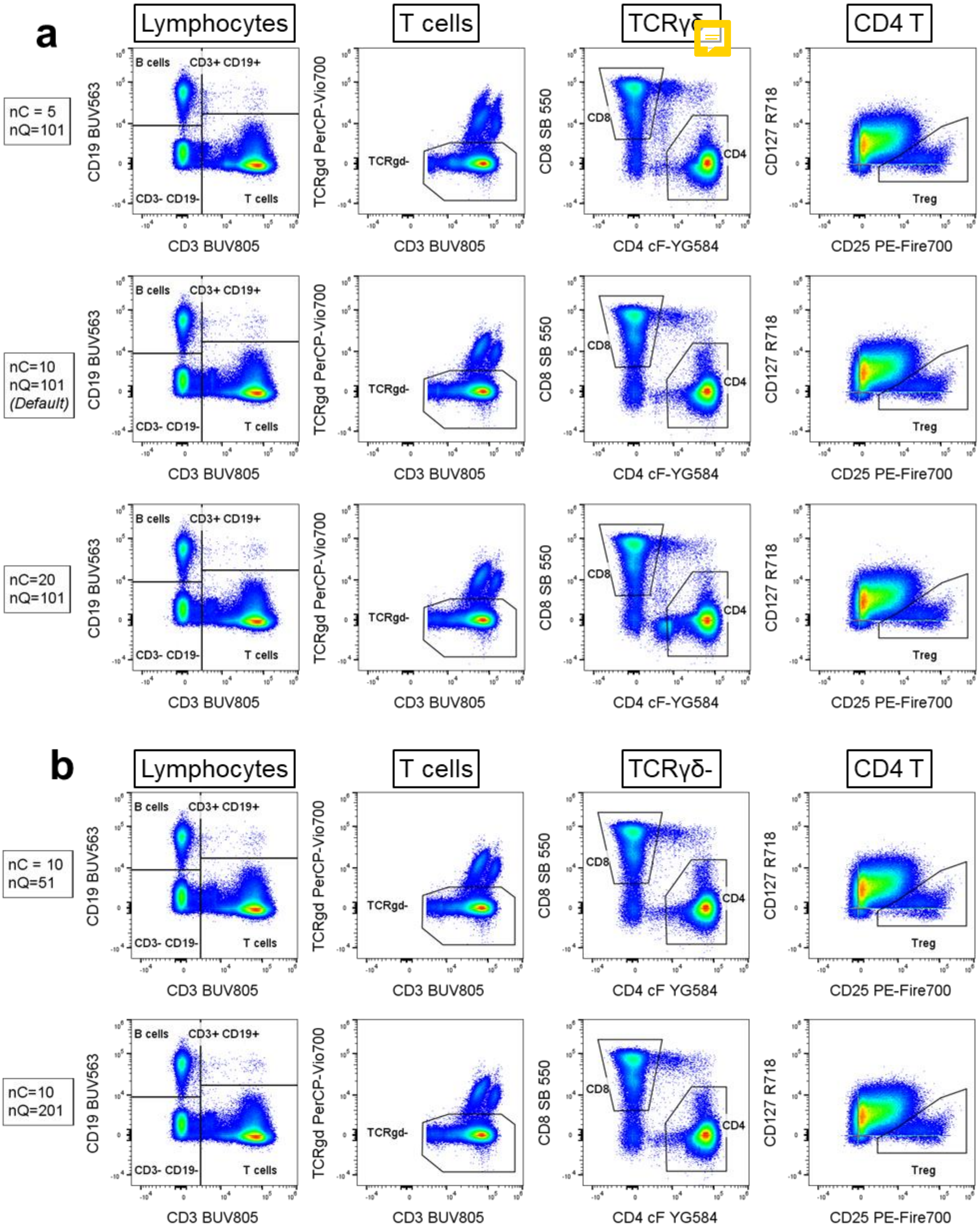

Supplementary figure 7: Adjustment of CytoNorm parameters does not reduce normalization artefacts. (**a**) Number of clusters (nC, default = 10) and (**b**) number of quartiles (nQ, default = 101) were adjusted.

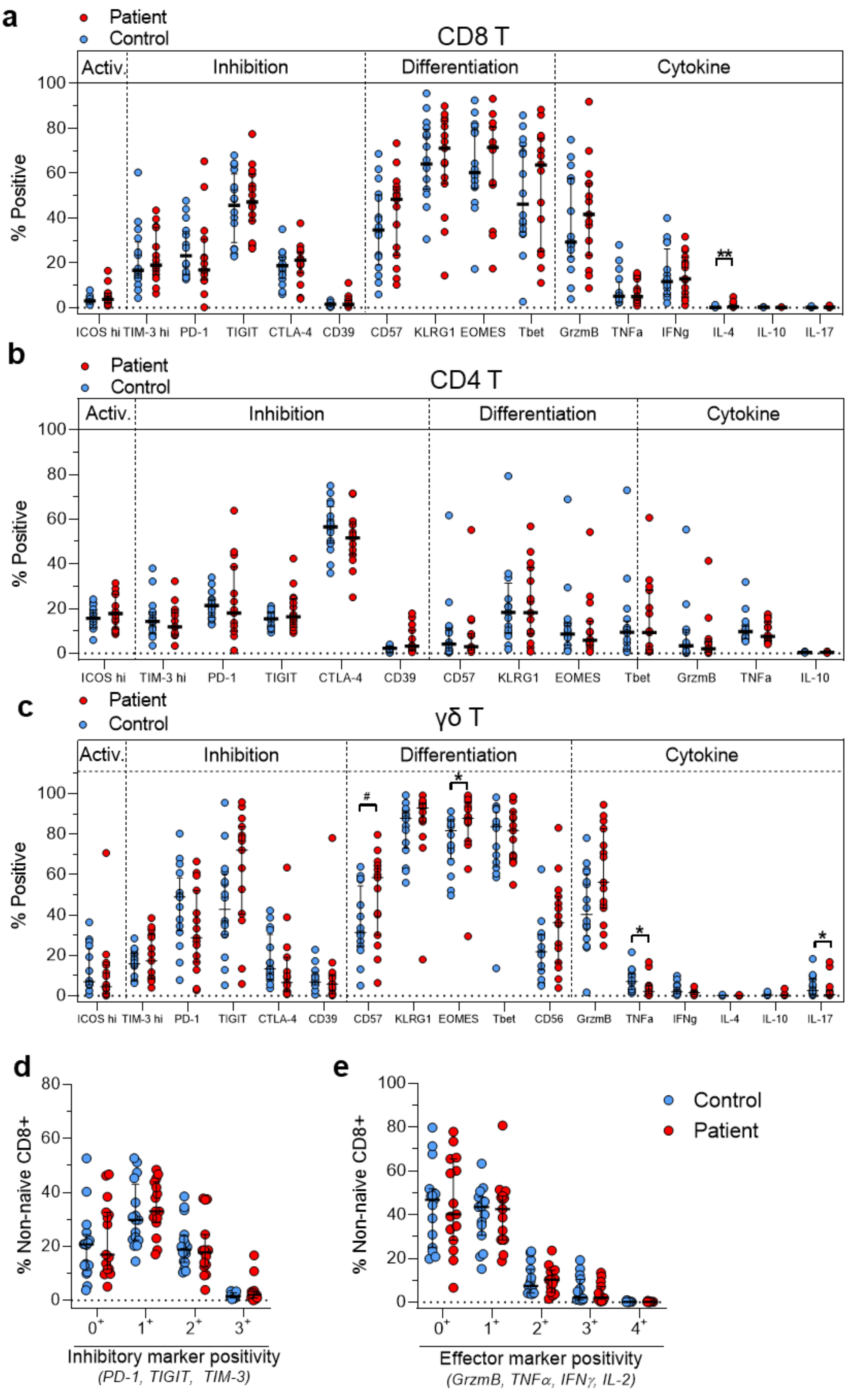

Supplementary figure 8: Activation, inhibition, differentiation, and cytokine marker expression across **(a)** total CD8$^+$ T, **(b)** non-Treg CD4$^+$ T, and **(c)** gamma-delta T (γδT) cells. **(d, e)** Boolean combinatorial gating of non-naive CD8$^+$ T cells for **(d)** inhibitory markers PD-1, TIM-3^{hi}, and TIGIT, and **(e)** effector polyfunctionality markers Granzyme B, TNFα, IFNγ, and IL-2. All graphs show median percent of parent population positive for individual markers or Boolean combinations in patients (n=15) and controls (n=16). Statistical tests use the unpaired non-parametric Mann-Whitney test. *$p < 0.05$, **$p < 0.01$, ***$p < 0.001$. Hash (#) symbols denote changes that did not reach significance after Bonferroni multiple comparisons correction.

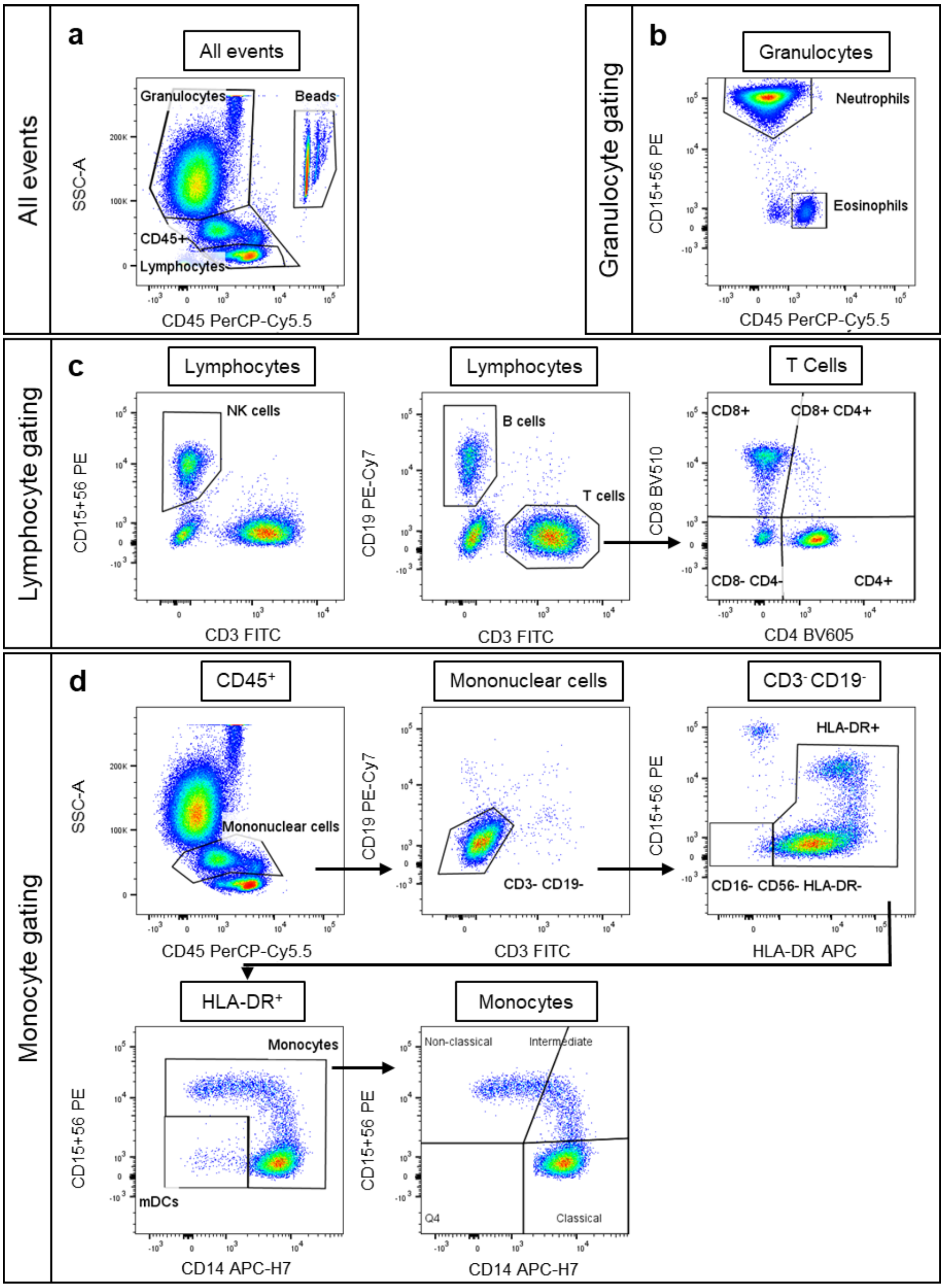

Supplementary figure 9: Gating scheme for TruCount analysis of whole blood. Whole blood was stained in a Lyse/No Wash protocol and analyzed on either an LSR II or FACSLyric (BD Biosciences). For FACSLyric acquisition (pictured), a CD45 threshold was set to exclude debris. **(a)** Major leucocyte populations were gated based on CD45 expression; granulocytes (CD45^{+} SSC^{hi}), lymphocytes (CD45^{+} SSC^{lo}) and TruCount beads (CD45^{hi}). **(b)** Granulocytes were separated into neutrophils (CD16^{+}) and eosinophils (CD16^{-}). **(c)** Lymphocytes were separated into NK cells (CD3^{-} CD16/56^{+}), B cells (CD3^{-} CD19^{+}) and T cells (CD3^{+} CD19^{-}). T cells were further characterized based on CD4 and CD8 expression. **(d)** Mononuclear cells were sequentially gated to remove contaminating cells and separated into myeloid dendritic cells (mDC; CD16/56^{-} CD14^{-} HLA-DR^{+}) and monocytes (HLA-DR^{+}). Monocytes were classified as classical (CD14^{+} CD16/56^{-}), intermediate (CD14^{+} CD16/56^{+}) and non-classical (CD14^{-} CD16/56^{+}). HLA-DR expression heatmap overlay was used to assist monocyte subset gating (not shown).
